# Pattern Completion in Multielement Event Engrams

**DOI:** 10.1016/j.cub.2014.03.012

**Published:** 2014-05-05

**Authors:** Aidan J. Horner, Neil Burgess

**Affiliations:** 1 UCL Institute of Cognitive Neuroscience, University College London, 17 Queen Square, London WC1N 3AR, UK; 2 UCL Institute of Neurology, University College London, Queen Square, London WC1 3BG, UK

## Abstract

Personally experienced events include multiple elements, such as locations, people, and objects. These events are thought to be stored in episodic memory as coherent representations [1] that allow the retrieval of all elements from a partial cue (“pattern completion” [2–6]). However, direct evidence for coherent multielement representations is lacking. Their presence would predict that retrieval of one element from an event should be dependent on retrieval of the other elements from that event. If we remember where we were, we should be more likely to remember who we met and what object they gave us. Here we provide evidence for this type of dependency in remembering three-element events. Dependency was seen when all three elements were encoded simultaneously, or when the three overlapping pairwise associations comprising an event were learned on separate trials. However, dependency was only seen in the separated encoding condition when all possible within-event associations were encoded. These results suggest that episodic memories are stored as coherent representations in which associations between all within-event elements allow retrieval via pattern completion. They also show that related experiences encountered at different times can be flexibly integrated into these coherent representations.

## Highlights

• Multielement events are stored as coherent engrams
• Retrievals of different associations from the same event are interdependent
• Independently encoded overlapping associations can form coherent event engrams
• Coherent engrams only form when all within-event associations are encoded

## Teaser

Horner and Burgess present evidence that the multiple associations comprising episodic events are bound within coherent “event engrams.” Related experiences encountered at different times can be flexible integrated into these engrams. Once formed, they are retrieved in an all-or-none manner by a process of pattern completion.

## Results

**Figure 1:**
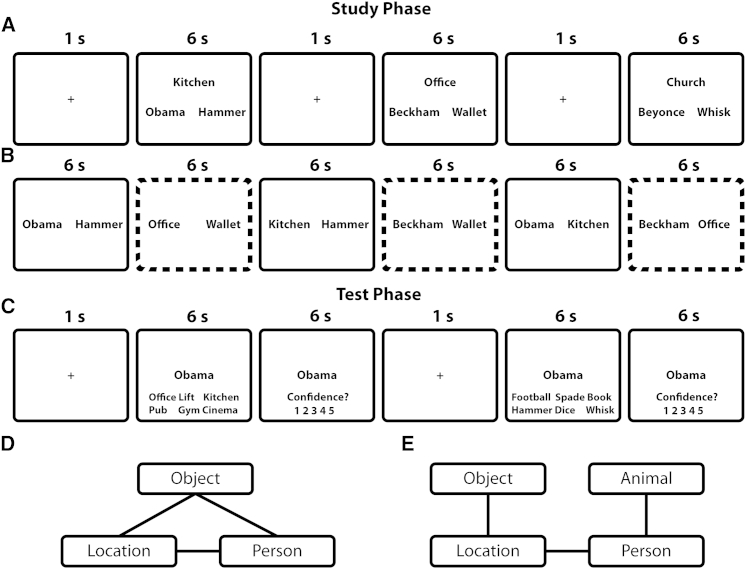
Trial Sequence for Study and Test Phases across Experiments (A) Trial sequence and timing for the Simultaneous condition of experiment 1. (B) Trial sequence (excluding 1 s fixation cross between encoding trials) for the Separated Closed-Loop and Separated Open-Loop conditions of experiments 1 and 2. Dotted lines are for illustrative purposes only (i.e., were not shown at encoding) to emphasize within-event pairs. Within-event pairs were not separated by a single intervening trial but had a mean of 36 intervening trials. (C) Trial sequence of cued-recognition during the test phase of experiments 1 and 2. Within-event pairs were not tested consecutively but were separated by a mean of 36 intervening trials. (D) Associative structure of the Simultaneous condition of experiment 1 and Separated Closed-Loop condition of experiments 1 and 2. (Note that half the Separated Closed-Loop events of experiment 2 were animal-location-person triads rather than object-location-person triads.) (E) Associative structure of the Separated Open-Loop condition of experiment 2.

Participants learned events composed of three or four elements (locations, people, and objects or animals) during a study phase (Figure 1 and Experimental Procedures). For example, for one event, they were presented with the words “kitchen,” “Barack Obama,” and “hammer” and required to imagine the three elements interacting. Using multielement events (as compared to simple pairwise associations) is critical to assess dependency. We can ask whether successful retrieval of one within-event association (e.g., retrieving location when cued with person) is dependent on retrieval of other associations from the same event (e.g., retrieving location when cued with object). During the test phase, each trial consisted of a cue (e.g., a location), with participants required to select the associated element (e.g., the person) among five other elements of the same type from different events. Each event was tested for all possible associations (e.g., location-person), in both directions, resulting in six retrieval trials per event.

We created contingency tables showing the dependency in performance when retrieving different associations from the same event (following [7–10]), e.g., the 2 × 2 table for retrieving the object or the person when cued by the location. The dependency measure reflects the proportion of events in which both associations were retrieved correctly or both incorrectly. By comparing this dependency to Independent and Dependent models of retrieval, we assessed within-event dependency for each participant, controlling for their accuracy and level of guessing (see Table 2, Experimental Procedures, and Supplemental Information available online). The Independent model predicts the contingency table corresponding to unrelated retrievals of different associations from the same event. The Dependent model predicts the contingency table corresponding to dependent retrieval of all associations from the same event. The models provide lower and upper bounds to the expected level of dependency.

In experiment 1, half of the “events” were seen in single encoding trials containing all three elements (the Simultaneous condition; Figure 1A); the other half were seen as three overlapping pairs of elements across three separate encoding trials (the Separated Closed-Loop condition; Figures 1B and 1D). For example, in the Separated Closed-Loop condition, we presented the location and person on one trial, then the location and object, and finally the person and object (with each encoding trial separated by trials from other unrelated events). All 72 trials, corresponding to 18 events in each condition, were presented in interleaved order. Memory for each association (six per event) was then tested in 216 interleaved trials (six-alternative forced-choice).

**Figure 2:**
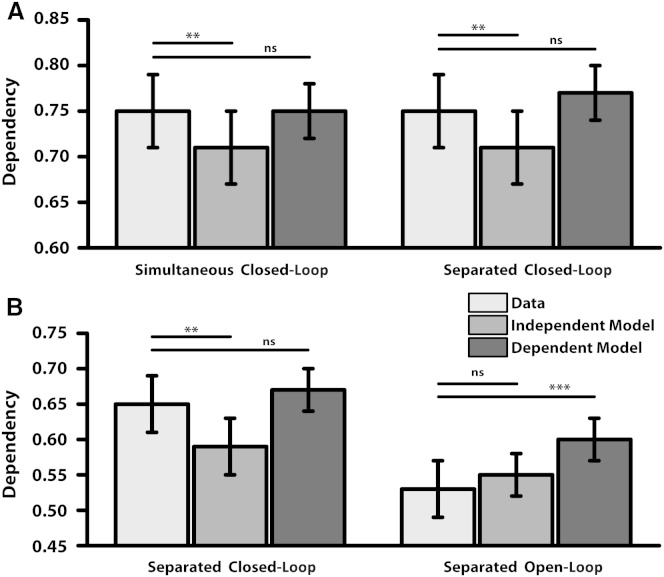
Dependency Analyses across Experiments 1 and 2 Dependency for the data, Independent model, and Dependent model across Simultaneous Closed-Loop and Separated Closed-Loop conditions of experiment 1 (A) and Separated Closed-Loop and Separated Open-Loop conditions of experiment 2 (B). Error bars represent ± 1 SE. ^∗∗∗^p < 0.001; ^∗∗^p < 0.01; ns, not significant.

Performance was good (76%; Table 1 and Supplemental Information). Contingency tables for the Simultaneous condition provided evidence for dependency (Figure 2A). Dependency exceeded the Independent model, *t*(15) = 2.95, p < 0.01, and did not differ from the Dependent model, *t*(15) = 0.81, p = 0.43 (see also [9]). The Separated Closed-Loop condition also showed greater dependency than the Independent model, *t*(15) = 3.14, p < 0.01, and did not differ from the Dependent model, *t*(15) = 1.32, p = 0.21. Importantly, the Simultaneous and Separated conditions showed similar dependency relative to their respective Independent models, *t*(15) = 0.25, p = 0.81.

Dependency comparable to the Dependent model was observed when the three elements of an “event” were presented simultaneously or in three separate pairwise encoding trials. These data suggest that episodic memories are stored as coherent representations and that related experiences encountered at different times can be integrated into these representations.

Experiment 2 aimed to replicate the finding of dependency in the Separated condition and to probe the conditions required for such dependency. In the Separated Closed-Loop condition, triads of the presented paired associates formed three-element events with an all-to-all or “closed-loop” associative structure (Figure 1D). Experiment 2 included triads of paired associates that formed four-element events (object-location-person-animal) with an open-loop associative structure (the Separated Open-Loop condition; Figure 1E). For example, participants would first encode location-object, then person-animal, and finally location-person. The 108 paired associates for 18 events from each condition were presented in interleaved trials (nine Closed-Loop events contained object-location-person, and nine contained animal-location-person).

As in experiment 1, performance was good (65%; Table 1 and Supplemental Information), and we saw dependency for the Separated Closed-Loop condition: dependency exceeded the Independent model, *t*(14) = 4.66, p < 0.001, and did not differ from the Dependent model, *t*(14) = 1.93, p = 0.07 (though we note a trend; Figure 2B). By contrast, we saw no evidence for dependency in the Separated Open-Loop condition: dependency did not differ from the Independent model, *t*(14) = 0.63, p = 0.54, and was significantly less than the Dependent model, *t*(14) = 4.78, p < 0.001. Importantly, the Closed- and Open-Loop conditions differed in dependency relative to their respective Independent models, *t*(14) = 3.48, p < 0.01.

Dependency is seen when an event is presented across separate encoding trials (the Separated Closed-Loop condition). However, this dependency was only seen when all possible within-event pairs were encoded (i.e., the Separated Open-Loop condition did not show dependency). This lack of dependency in the Open-Loop condition was replicated, despite changing the order of the encoded pairs (experiment S1), and using paired associates from three-element events with an open-loop structure (where only two of the three possible associations were encoded; experiment S2). Thus, coherent event representations can be constructed across multiple encoding trials, but this depends on the associative structure presented, with a closed loop of three associations producing dependency, but not an open chain of three (or two) associations.

## Discussion

Episodic memories are thought to be stored as coherent representations of the multiple elements comprising an event (“event engrams”). We found dependency in the retrieval of different elements of the same event, even when the event was formed from overlapping pairwise associations presented across separate trials, but only when all possible pairwise associations in the event were presented.

The associative structure of events has been investigated using partial cuing techniques [11, 12]; however, this approach does not address within-event dependency or variation across events. Dependency has also been assessed in memory for subordinate features (e.g., location on a screen and font size) of single elements (e.g., words) [13–15]. However, here we are interested in event memory, the binding of independently represented multimodal elements into coherent representations. Furthermore, these previous studies concerned “events” encoded on single trials and did not assess dependency for overlapping but independently encoded pairwise associations (i.e., our Separated conditions).

Dependency for events formed from simultaneously presented elements could reflect trial-by-trial modulation of attention (see [9]), since attention at encoding can modulate memory performance (e.g., [16–18]). However, we saw similar dependency for events formed from overlapping pairs of elements presented over different trials, ruling out an attentional explanation. These results also challenge models in which item information is associated via a time-varying context signal (e.g., [19, 20]), as its time-varying nature would cause independence in the Separated Closed-Loop condition. If a common “context” representation mediates within-event associations, it must predominantly comprise the within-event elements themselves. This forces us toward a mechanism in which within-event associations can be encoded independently but are retrieved in a dependent manner.

We suggest that dependency results from the associative structure of the “event.” If all possible within-even pairs are encoded, a partial cue can cause retrieval of all within-event elements (regardless of whether they are being tested or not). This pattern completion process is thought to be a core function of the hippocampus [2–6], a region critical for episodic memory [21–23]. We suppose that hippocampal neurons selectively code for individual elements of any given event, consistent with “place cells” in rat [24] or human [25] hippocampus that represent specific locations and single neurons in the human hippocampus representing specific famous people [26].

In this view, it is the closed-loop structure of within-event associations that constitutes a coherent representation and allows pattern completion. This can be captured by simple autoassociative memory models (e.g., [2, 5, 6, 27]), in which reactivation of an individual element depends on the strengths of association between all elements within the event. In this case, retrieval performance on any one trial will reflect the strength of all within-event associations. The Dependent model captures this by assuming that performance on a retrieval question reflects the mean performance on the other questions regarding that event (the episodic factor *E*; see Experimental Procedures and Supplemental Information). Within this account, the lack of dependency in the Open-Loop condition reflects an absence of pattern completion: the only route for the cue to reactivate the target is via the cue-target association itself.

It is possible that pattern completion occurs at encoding (as well as at retrieval), allowing simultaneous encoding of all preceding within-event associations, which could introduce dependency in their strengths (see [28] for a related proposal). For example, when encoding the last pair (e.g., A-C), both the A-B and C-B associations could be retrieved, possibly leading to explicit imagery of all three elements. Note that this account still relies on the presence of pattern completion, albeit at encoding, and as such is constrained by the associative structure of the event: only occurring for closed-loop structures.

Our results have implications for how coherent event representations are formed from continuous experience. Features of the incoming stream of information can form contextual boundaries, segmenting our perception of the world into discrete events (e.g., [29, 30]) and influencing what information is bound within an event engram (e.g., [31, 32]). The presence of an “event boundary” can trigger the binding of all elements experienced in the preceding context, a process in which the hippocampus has been implicated (e.g., [33]). However, the presence of such an event boundary, demarcating a contiguous segment of time, may not be a necessary precondition for such binding to occur. Our results suggest that dependency can result from the associative structure of the related elements, rather than necessarily depending on their having been presented within the same context (cf. [19, 20]).

Although dependency was not seen for open-loop associative structures, presenting pairs A-B and A-C in experiment S2 led to above-chance performance for the nonencoded pairs (B-C), suggesting the presence of a weak association of nonencoded pairs, perhaps due to reactivation of A-B on presentation of A-C [28]. This association was presumably too weak to result in strong pattern completion of all three elements at retrieval, as dependency was not seen. Perhaps dependency for open-loop structures would be seen if nonencoded associations were sufficiently strengthened, by repetition or offline consolidation [34–37], potentially allowing for generalization across elements that have not been directly associated, a process that may also be mediated by the hippocampus [38–40].

The formation of integrated closed-loop structures over time might relate to the concept of “schema” [11, 41–43], consolidated memory structures that allow the integration of new related information. Our focus was on episodic memory and single presentations of memoranda, rather than long-term learning of statistical relationships over multiple presentations, which is the traditional focus of semantic learning and systems consolidation [2, 5, 44, 45] and in which “chunks” can be formed from higher-order relationships [46, 47]. Nonetheless, our “associative structures” may represent building blocks from which schema can be built. Although the exact relationships between traditionally defined “events,” our separately encoded “associative structures,” and “schema” are currently unclear, our approach presents opportunities for bridging the fields of episodic memory and the segmentation, generalization, and consolidation of experience.

### Conclusions

Theories of episodic memory propose the existence of coherent event representations, allowing retrieval of all event elements via pattern completion. Here we present evidence that such event engrams exist and are built from multiple overlapping associations that can be encoded independently. Performance in retrieving any within-event association is related to performance for retrieving other associations from the same event. We suggest this dependency results from a retrieval-related pattern completion process, requiring the presence of a closed-loop structure of associations between within-event elements. Our results shed light on how the episodic memory system can rapidly incorporate new information into associative structures, and how multielement events are retrieved through a process of pattern completion.

## Experimental Procedures

### Participants

All experiments were approved by the University College London Research Ethics Committee (NB/PWB/26102011a), and all participants gave informed consent (see Supplemental Information for participant details).

### Materials

Stimuli were 36 locations (e.g., a swimming pool), famous personalities (e.g., David Cameron), common objects (e.g., a bicycle; see [9]), and animals (e.g., a dog).

### Procedure

Experiments consisted of single study and test phases. At study, triads (for the Simultaneous condition) or pairs (for the Separated conditions) of elements were serially presented (Figure 1). Triads/pairs were presented for 6 s, as words, and participants were required to imagine the elements on the screen “interacting in a meaningful way as vividly as possible.” Experiment 1 presented triads and pairs; experiment 2 presented only pairs (see Supplemental Information).

At test, participants were presented with an element and had to choose the associated element from six alternatives (Figure 1). All “events” were tested with every cue-test pair (e.g., cue: location, test: object), resulting in six cued-recognition trials per event. One of the test items was the element associated with the cue; the other five were elements of the same category (e.g., objects) randomly selected from other events (regardless of condition). Participants were required to respond as accurately as possible within 6 s with a key press and to rate their confidence on a scale of 1 to 5.

### Assessing Dependency

We created 2 × 2 contingency tables of each participant’s performance for specific pairs of associations across events, including tables for retrieving two elements (e.g., person and object) when cued by the remaining element (e.g., location; “A_B_A_C_” analyses) and for retrieving one element (e.g., location) when cued by its associated elements (e.g., person and object; “B_A_C_A_” analyses). This resulted in six 2 × 2 tables per participant per condition in experiments 1 and S2, one for each element (item type) and analysis type (A_B_A_C_ or B_A_C_A_), and four tables per participant per condition in experiments 2 and S1 (testing the four pairs of associations common to both Open- and Closed-Loop conditions; see Supplemental Information).

To assess dependency, we took the proportion of events in which both associations were either correctly or incorrectly retrieved and averaged this measure across the contingency tables for a given condition. We also calculated Yule’s Q measure of dependency (see [8, 10, 48]) for the data for all experiments (see Supplemental Information).

For each contingency table, we created predicted tables corresponding to “Independent” and “Dependent” models of retrieval (see Table 2, Supplemental Information, and [9] for details). The Independent model predicts the dependency corresponding to the participant’s mean level of performance for the two associations across events. In the Dependent model, the predicted retrieval performance for a given question is adjusted by the mean performance over other questions for that event (the episodic factor *E*). It predicts the maximal level of dependency, given the participant’s mean level of performance for the two associations, their overall level of guessing, and the amount of variance in their overall performance across events. The Independent and Dependent models serve as theoretical lower and upper bounds for comparison to the level of dependency in the data, for each participant in each condition.

## Figures and Tables

**Table 1:** Memory Performance across Experiments 1 and 2

	Cue Type	Retrieved Type			
		Location	Person	Object	Animal
**Experiment 1**					

Sim. Closed	Location	NA	0.80 (0.20)	0.72 (0.22)	NA
	Person	0.79 (0.23)	NA	0.76 (0.22)	NA
	Object	0.74 (0.23)	0.76 (0.20)	NA	NA
Sep. Closed	Location	NA	0.77 (0.18)	0.78 (0.20)	NA
	Person	0.77 (0.19)	NA	0.76 (0.26)	NA
	Object	0.77 (0.22)	0.79 (0.18)	NA	NA

**Experiment 2**					

Sep. Closed	Location	NA	0.64 (0.19)	0.68 (0.22)	0.80 (0.15)
	Person	0.60 (0.21)	NA	0.69 (0.19)	0.61 (0.14)
	Object	0.71 (0.18)	0.67 (0.22)	NA	NA
	Animal	0.70 (0.17)	0.64 (0.19)	NA	NA
Sep. Open	Location	NA	0.51 (0.22)	0.76 (0.20)	NA
	Person	0.51 (0.24)	NA	NA	0.58 (0.15)
	Object	0.75 (0.19)	NA	NA	NA
	Animal	NA	0.64 (0.18)	NA	NA

Proportion correct cued recognition (and SD) for each retrieved type (i.e., the element the participants were tested on; columns) and each cue type (i.e., the element the participants were cued with; rows) across the Simultaneous Closed-Loop (Sim. Closed) and Separated Closed-Loop (Sep. Closed) conditions of experiment 1 and Separated Closed-Loop and Separated Open-Loop (Sep. Open) conditions of experiment 2.

**Table 2:** The Independent and Dependent Models

Retrieval of Element (C)	Retrieval of Element (B)	
	Correct (*P*_*AB*_)	Incorrect (1 − *P*_*AB*_)
**Independent Model**		

Correct (*P*_*AC*_)	∑ _*i*=1_^*N*^*P*_*AB*_*P*_*AC*_	∑ _*i*=1_^*N*^*P*_*AC*_ (1 *− P*_*AB*_)
Incorrect (1 *− P*_*AC*_)	∑ _*i*=1_^*N*^*P*_*AB*_ (1 *− P*_*AC*_)	∑ _*i*=1_^*N*^(1 *− P*_*AB*_)(1 *− P*_*AC*_)

**Dependent Model**		

Correct (*P*_*AC*_)	∑ _*i*=1_^*N*^*Ṕ*^*i*^_A*B*_*Ṕ*^*i*^_*AC*_	∑ _*i*=1_^*N*^*Ṕ*^*i*^_*AC*_ (1 *− Ṕ*^*i*^_*AB*_)
Incorrect (1 *− P*_*AC*_)	∑_*i*=1_^*N*^*Ṕ*^*i*^_*AB*_ (1 *− Ṕ*^*i*^_*AC*_)	∑ _*i*=1_^*N*^(1 *− Ṕ*^*i*^_*AB*_)(1 *− Ṕ*^*i*^_*AC*_)

Contingency tables for the Independent and Dependent models, giving the frequency (over events) of the four combinations of correct or incorrect retrieval of elements B and C when cued by element A. The Dependent model replaces the probability of correctly recalling B when cued by A (across all events; *P*_*AB*_) with *Ṕ*^*i*^_*AB*_*= E*^*i*^_*AB*_(*P*_*AB*_ − *P*_*G*_/*c*) *+ P*_*G*_/*c*, where the episodic factor *E*^*i*^_*AB*_ reflects performance on event *i* relative to other events (based on retrievals other than B and C cued by A), *P*_*G*_ is the probability of guessing, and *c* = 6 is the number of choices in a test trial. *P*_*AC*_ is replaced similarly (see Supplemental Information for details). The Dependent model equates to the Independent model if the episodic factors are set to 1.

## Supplemental Information

Document S1. Supplemental Results, Figure S1, and Supplemental Experimental Procedures

Document S2. Article plus Supplemental Information
