# Supplementary material for: Pattern Completion in Multielement Event Engrams
Source: Curr Biol. 2014 May 5;24(9):988–92. doi: 10.1016/j.cub.2014.03.012 (PMC4012134; doi:10.1016/j.cub.2014.03.012)
Supplement: Document S1. Supplemental Results, Figure S1, and Supplemental Experimental Procedures [file mmc1.pdf]

Current Biology, Volume *24*

Supplemental Information

Pattern Completion in Multielement Event Engrams

Aidan J. Horner and Neil Burgess

Supplemental Information

Supplemental Results

Further analyses of Experiments 1 & 2

Cued-recognition performance

Experiment 1

A 2x3 (Simultaneous vs. Separated x Cue-type) within-subjects ANOVA of retrieval performance, collapsed across Retrieved-type, failed to reveal any significant effects, *F*'s<1.07, p's>.34, η_P^{2}'s<.07. A similar 2x3 (Simultaneous vs. Separated x Retrieved-type) ANOVA, collapsed across Cue-type, also failed to reveal any significant effects, *F*'s<1.35, p's>.27, η_P^{2}'s<.08. Thus, no differences in performance were seen across the three item types (location, people and objects), either as the cue-type or retrieved-type. Further, no difference in performance was seen between the Simultaneous and Separated conditions.

Experiment 2

A 2x4 (Closed-loop vs. Open-loop x Cue-type) within-subjects ANOVA of retrieval performance, collapsed across Retrieved-type, revealed a Closed-loop vs. Open-loop x Cue-type interaction, $F(1.6, 22.1) = 4.58$, $p<.05$, $\eta_P^2=.25$, and a main effect of Cue-type, $F(2.1, 29.5) = 6.56$, $p<.001$, $\eta_P^2=.32$. Despite a trend, no significant difference was seen between Closed-loop vs. Open-loop, $F(1, 14) = 3.86$, $p=.07$, $\eta_P^2=.22$. The interaction was characterised by an effect of Cue-type in the Open-loop condition, $F(2.0, 27.4) = 9.03$, $p<.01$, $\eta_P^2=.39$, with better performance when cued by an object relative to the other three elements, but no Cue-type effect in the Closed-loop condition, $F(1.9, 26.2) = 1.67$, $p=.21$, $\eta_P^2=.11$.

A similar 2x4 (Closed-loop vs. Open-loop x Retrieved-type) ANOVA, collapsed across Cue-type, revealed a similar Closed-loop vs. Open-loop x Cue-type interaction, $F(3.0, 41.38) = 12.24$, $p<.001$, $\eta_P^2=.47$, as well as main effects of Retrieved-type, $F(2.1, 30.1) = 5.68$, $p<.01$, $\eta_P^2=.29$, and of Closed-loop vs. Open-loop, $F(1, 14) = 6.34$, $p<.05$, $\eta_P^2=.31$. Again, the interaction was characterised by an effect of Retrieved-type in the Open-loop condition, $F(2.5, 35.3) = 11.04$, $p<.001$, $\eta_P^2=.44$, with better performance when retrieving the object relative to the other three elements, but no Retrieved-type effect in the Closed-loop condition, $F(1.8, 24.9) = 1.77$, $p=.19$, $\eta_P^2=.11$. To summarise, performance was better in the Closed-loop than Open-loop condition, though this only reached significance in the analysis across Retrieved-type, and performance was more variable across the four elements in the Open-loop than Closed-loop condition.

The differences in memory performance across the four item-types (locations, people, objects and animals) in the Open-loop but not Closed-loop condition suggest an underlying difference in memory strength across item types, with better memory for objects. However, when dependency is seen (i.e., in the Closed-loop condition), accuracy becomes more similar across item types. This finding is consistent with the presence of pattern completion in the Closed-loop condition, in which performance reflects all within-event associations rather than just the association between the specific elements tested.

Analysis of dependency across Analysis-type and Item-type

Experiment 1

A 2x2x3 (Simultaneous vs. Separated x Analysis-type x Item-type) ANOVA on dependency of the observed data failed to reveal any main effects or an interaction (F's<2.23, p's$>.15$,

η_P^2's<.13). Analysis-type refers to 'A_BA_C' versus 'B_AC_A' analyses (see Experimental Procedures). Item-type refers to the type of the common element in the dependency analysis (i.e., the element referred to as A in the above description of analysis-type).

Experiment 2

A 2x2x2 (Closed-loop vs. Open-loop x Analysis-type x Item-type) ANOVA on dependency of the observed data revealed a main effect of Closed-loop vs. Open-loop, $F(1, 14) = 12.87$, $p<.01$, $\eta_P^2=.48$, reflecting the difference in dependency between the two conditions (but potentially also reflecting accuracy differences, which are controlled for by the comparisons to Independent and Dependent models in the main text). No further main effects or interactions reached significance, F's<1.7, p's>.22, η_P^2's<.11.

Table S1, related to Figure 2. Mean dependency (D) (and standard deviation) for the A_BA_C and B_AC_A analyses for the observed data across the Simultaneous Closed-loop (Sim. Closed) and Separated Closed-loop (Sep. Closed) conditions of Experiment 1 and Separated Closed-loop (Sep. Closed) and Separated Open-loop (Sep. Open) conditions of Experiment 2.

Analysis:	A_BA_C			B_AC_A		
Item-type:	Location	Person	Object	Location	Person	Object
Experiment 1						
Sim. Closed	.76 (.14)	.77 (.17)	.74 (.16)	.76 (.17)	.71 (.21)	.74 (.15)
Sep. Closed	.79 (.17)	.77 (.15)	.73 (.19)	.75 (.18)	.74 (.18)	.75 (.18)
Experiment 2						
Sep. Closed	.69 (.16)	.61 (.18)	n/a	.63 (.13)	.68 (.15)	n/a
Sep. Open	.55 (.17)	.54 (.17)	n/a	.53 (.20)	.53 (.13)	n/a

Analysis of encoding order in the Separated Closed-loop condition

In Horner & Burgess (S1), we raised the possibility that dependency could change across the 6 retrieval trials per event. Interestingly, although we saw an increase in accuracy from retrieval trial 1-6, we saw no evidence for any increase in dependency when pairing the 1st and 2nd, 3rd and 4th and 5th and 6th retrieval trials for each event. In fact, the analyses raised the possibility of a small decrease in dependency, once the overall increase in accuracy was taken into account. In previous studies, an 'event' was presented in a single trial with all elements shown on the screen at the same time (equivalent to the Simultaneous condition of experiment 1). Given that we introduced a 'Separated' condition for the current studies, we can ask a similar question at encoding. Does dependency differ as a function of when each pairwise association was encoded? To do this, we paired up retrieval trials for the 1st vs. 2nd encoded pair, 2nd vs. 3rd encoded pair and 1st vs. 3rd encoded pair of each event, and conducted the same dependency analyses as in the main text. Note, this analysis was only performed on the Separated Closed-loop conditions of experiments 1 and 2 (as these were the Separated conditions that showed evidence for dependency).

Experiment 1

A one-way ANOVA (1st vs. 2nd vs. 3rd encoded pair) analysing cued-recognition performance showed a significant main effect, $F(1.7, 25.6) = 3.63$, $p<.05$, $\eta_P^2=.20$, revealing greater performance for 1st (81%), than 2nd (76%) and 3rd (75%) encoded pairs. However, dependency does not appear to be affected by encoding order. A one-way ANOVA comparing the raw dependency measure for 1st vs. 2nd, 2nd vs. 3rd and 1st vs. 3rd encoded pairs was not significant, $F(1.8, 27.5) = .93$, $p=.41$, $\eta_P^2=.06$. A similar ANOVA on the difference between dependency in the data and the Independent model (i.e., controlling for

accuracy differences) for the same 3 pairings was also not significant, $F(1.7, 26.8) = .84$, $p=.43$, $\eta_P^2=.05$. Thus, we could find no evidence for differences in dependency as a function of the order of encoded pairs.

Experiment 2

A one-way ANOVA (1st vs. 2nd vs. 3rd encoded pair) analysing cued-recognition performance showed a significant main effect, $F(1.8, 25.7) = 6.91$, $p<.01$, $\eta_P^2=.33$, revealing greater performance for 1st (72%), than 2nd (66%) and 3rd (62%) encoded pairs. Again, dependency does not appear to be affected by encoding order. A one-way ANOVA comparing the raw dependency measure for 1st vs. 2nd, 2nd vs. 3rd and 1st vs. 3rd encoded pairs was not significant, $F(1.5, 21.5) = 2.05$, $p=.16$, $\eta_P^2=.13$. A similar ANOVA on the difference between dependency in the data and the Independent model (i.e., controlling for accuracy differences) for the same 3 pairings did reach significance, $F(1.9, 27.3) = 3.79$, $p<.05$, $\eta_P^2=.21$, with less dependency for the 1st vs. 2nd pair (dependency for Data – Independent model = 0.01) than 2nd vs. 3rd (0.06) or 1st vs. 3rd (0.04).

Although this difference was unexpected, a full 2x3 mixed ANOVA across experiments 1 and 2, comparing the difference between the data and Independent model across the 3 pairings failed to reveal a main effect of encoding pair, $F(1.9, 55.3) = 1.35$, $p=.27$, $\eta_P^2=.05$, with no interaction between this factor and Experiment, $F(1.9, 27.3) = 2.78$, $p=.07$, $\eta_P^2=.08$ (though we note a trend). Thus, across experiments we could not find consistent evidence for differences in dependency as a function of the order of encoded pairs.

Analysis of reaction times

Though not central to our main hypotheses, it is conceivable that differences in reaction times (RTs) between our conditions of interest may have contributed to differences in dependency. For example, perhaps longer RTs in one condition might allow participants to explicitly retrieve all associated elements of an event, leading to increases in dependency for that condition (i.e., such a finding might help to interpret our observations of dependency). In experiment 1, no difference was seen between the Simultaneous (mean: 3270msecs) and Separated Closed-loop (3233msecs) conditions, $t(15) = .53$, $p=.60$. In experiment 2, no difference was seen between the Separated Closed-loop (3550msecs) and Separated Open-loop (3668msecs) conditions, $t(14) = 1.46$, $p=.17$. Thus, we could find no evidence for RT differences at retrieval between our main experimental conditions.

Supplemental Experiments S1 & S2

Experiment S1 – Closed- vs. Open-loop 4-item events

The Open-loop condition of experiment 2 presented the paired associates for each event in a specific order. For example, if the location-object pair was encoded first, the 2nd pair would have been person-animal and the last pair was always location-person (see Supplemental Experimental Procedures). Thus, the 2nd encoded pair never overlapped with the 1st encoded pair. This was not the case in the Closed-loop condition, where the 2nd encoded pair by necessity had to overlap with the 1st encoded pair. Experiment S1 controlled for this possible encoding order issue. Regardless of the 1st encoded pair, the 2nd encoded pair in the Open-loop condition always overlapped with the 1st pair (see

Supplemental Experimental Procedures). For example, if participants first learned location-object, the next encoded pair would be location-person and then person-animal. Thus, any lack of dependency in the Separated Open-loop condition of experiment S1 could not result from the fact that the first two learned pairs for any 'event' were completely unrelated (and only subsequently connected by the final encoded pair).

Cued-recognition performance

Performance was well above chance for both the Closed-loop (61%) and Open-loop (62%) conditions. A 2x4 (Closed-loop vs. Open-loop x Cue-type) within-subjects ANOVA on retrieval performance, collapsed across Retrieved-type, revealed a Closed-loop vs. Open-loop x Cue-type interaction, $F(2.6, 34.2) = 7.43$, $p<.01$, $\eta_P^2=.36$, as well as a main effect of Cue-type, $F(2.7, 35.5) = 3.49$, $p<.05$, $\eta_P^2=.21$. No main effect of Closed- vs. Open-loop was seen, $F(1, 13) = .23$, $p=.64$, $\eta_P^2=.02$. The interaction was characterised by a larger effect of Cue-type in the Open-loop condition, $F(2.6, 33.5) = 9.21$, $p<.001$, $\eta_P^2=.42$, relative to the Closed-loop condition, $F(2.8, 36.1) = 4.42$, $p<.05$, $\eta_P^2=.25$, with better performance when cued by an object relative to the other three elements in the Open-loop condition. A similar 2x4 (2x4 (Closed-loop vs. Open-loop x Retrieved-type) ANOVA, collapsed across Cue-type, failed to reveal any significant effects or interactions, F's<2.2, p's>.13. As in experiment 2, performance was more variable across item-type in the Open-loop than Closed-loop condition (albeit only for the Cue-type analysis in experiment S1).

Dependency analysis

A 2x2x2 (Closed-loop vs. Open-loop x Analysis-type x Item-type) ANOVA on dependency of the observed data revealed a main effect of Closed-loop vs. Open-loop, $F(1, 13) = 18.62$,

p<.001, η_P^2=.59 (as in Experiment 2). Apart from a trend towards a Closed- vs. Open-loop x Item-type interaction, $F(1, 13) = 3.49$, p=.09, η_P^2=.21, no further main effects or interactions were seen, *F*'s<1.3, p's>.26. As in experiments 1 & 2, we collapsed across Analysis-type and Item-type for comparisons between the data and the models.

Dependency for the data, Independent model and Dependent model for Closed-loop and Open-loop events is shown in Figure S1. For the Closed-loop events, dependency was greater than the Independent model, $t(13) = 2.85$, p<.05, and did not differ from the Dependent model, $t(13) = 1.41$, p=.18. For the Open-loop events, dependency did not differ from the Independent model, $t(13) = 1.89$, p=.08 (though we note a trend in the opposite direction, i.e., less dependency in the data than the Independent model), but was less than the Dependent model, $t(13) = 8.35$, p<.001. Importantly, the difference in dependency between the data and Independent model (Data – Independent model) was greater in the Closed-loop than Open-loop condition, $t(13) = 3.37$, p<.01. Experiment S1 therefore replicates the results of experiment 2. An open-loop associative structure shows less dependency than a closed-loop structure, even when we change the encoding order to ensure all 2nd encoded pairs overlap with 1st encoded pairs regardless of condition.

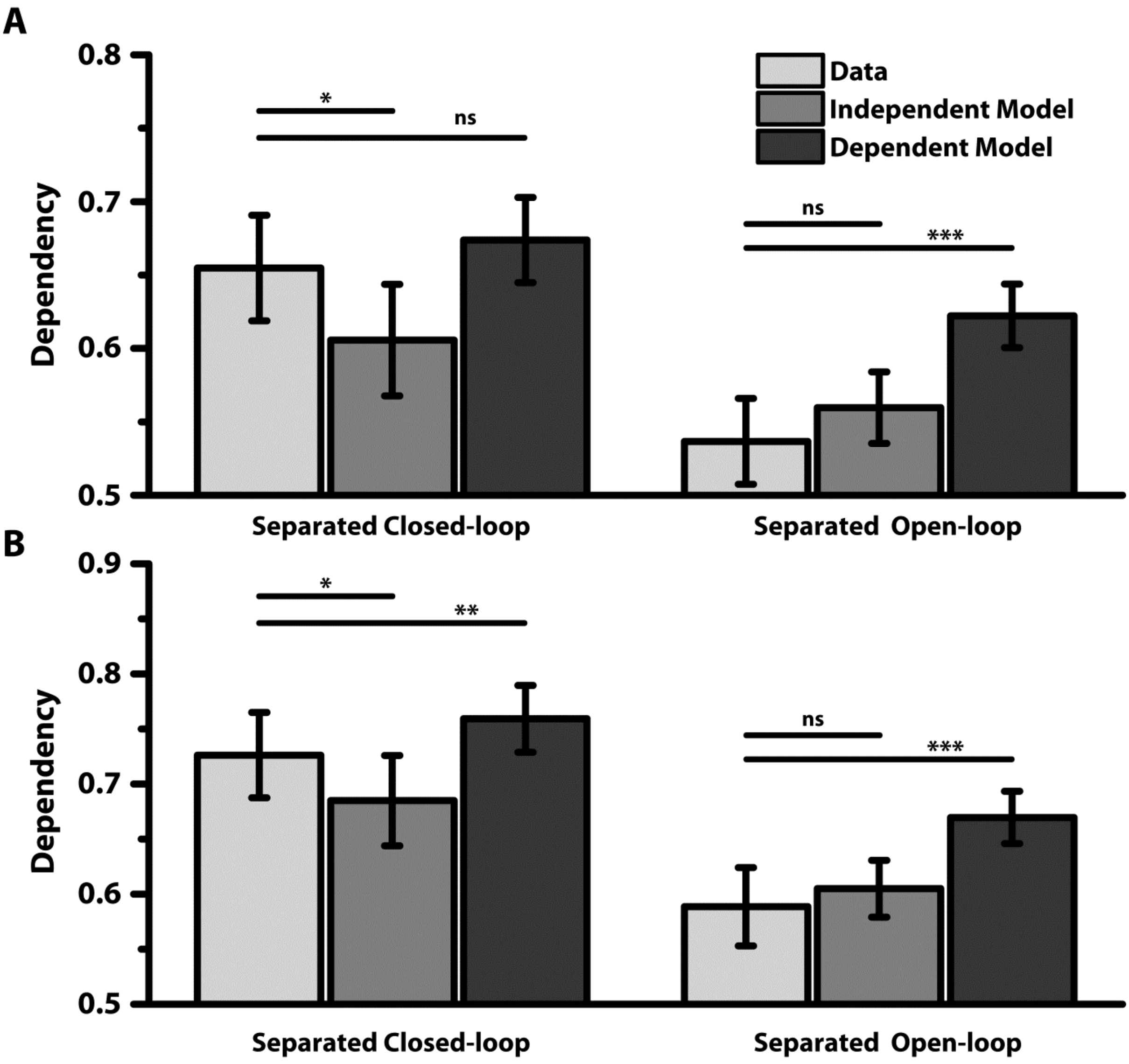

Figure S1, related to Figure 2. Dependency for the data, Independent model and Dependent model across the Separated Closed-loop and Separated Open-loop conditions of (a) experiment S1 and (b) experiment S2. Error bars represent +/- 1 standard error; *** p<.001; ** p<.01; * p<.05; ns = not significant.

Experiment S2 – Closed- vs. Open-loop 3-item events

The Open-loop condition of experiments 2 & S1 included a fourth element in the associative structure of the event engram. This was done to equate the number of associations within the engram, as well as familiarity with the location and person elements of the events, between the Open-loop and Closed-loop conditions. However, it is possible that it was the

fourth element, as opposed to the open-loop structure, of the event engram that resulted in the decrease in dependency seen in experiment 2 and S1. To rule out this possibility, we ran a fourth experiment where participants were only presented with 3-element events (location-person-object triads).

In a between-subject manipulation, half the participants were presented with all three possible pairs during encoding (across separate encoding trials; the Separated Closed-loop condition) and half the participants were presented with only two out of the three possible pairs (the Separated Open-loop condition). For example, they might be shown the location-person and person-object pair, but not the location-object pair, at encoding (see Supplemental Experimental Procedures). Therefore, the Open-loop condition of experiment S2 controls for the number of elements within an 'event', but not the number of associations, whereas the Open-loop condition of experiment 2 and S1 controlled for the number of associations, but not the number of elements. If the decrease in dependency in experiment 2 and S1 was due to the open-loop associative structure of the event engram (as opposed to the number of elements within an event), we should see a similar decrease in dependency when only two out of the three possible pairs of a triad are presented.

Cued-recognition performance

Performance was well above chance for both the Closed-loop (75%) and Open-loop (66%) conditions. A 2x3 (Closed-loop vs. Open-loop x Cue-type) mixed ANOVA failed to reveal any significant effects on performance, F's<1.70, p's>.20, η_P^2's<.06. A similar ANOVA across Retrieved-type showed a trend for a main effect of Retrieved-type, $F(1.9, 53.2) = 3.16$, $p=.05$, $\eta_P^2=.10$. This effect appeared to be driven by greater accuracy when retrieving the Person relative to Locations and Objects. No further significant effects were seen, F's<1.63,

p's>.21, η_P^2's<.06. Thus, no difference was seen in accuracy between the Closed-loop and Open-loop group.

Dependency analysis

A 2x2x3 (Closed-loop vs. Open-loop x Analysis-type x Item-type) mixed ANOVA on dependency of the observed data revealed a main effect of Closed-loop vs. Open-loop, $F(1, 28) = 6.08$, $p<.05$, $\eta_P^2=.18$, with greater dependency in the Closed-loop than Open-loop condition. Apart from a Closed-loop vs. Open-loop x Item-type interaction, $F(1.8, 49.7) = 4.79$, $p<.05$, $\eta_P^2=.15$, no further significant effects were seen, *F*'s<2.70, p's>.08, η_P^2's<.09. As in experiments 1 & 2, we collapsed across Analysis-type and Item-type for comparisons between the data and the models.

Dependency for the data, Independent model and Dependent model for Closed-loop and Open-loop events is shown in Figure S1. For the Closed-loop events, dependency was greater than the Independent model, $t(13) = 2.76$, $p<.05$, replicating the findings of experiments 1,2 & S1. As in experiment 2 & S1, we saw no difference between the data and Independent model for the Open-loop events, $t(15) = .94$, $p=.36$. Importantly, the difference between the data and Independent model for the Closed-loop group was greater than for the Open-loop group, $t(28) = 2.41$, $p<.05$. Finally, both the Closed-loop, $t(13) = 3.29$, $p<.01$, and Open-loop, $t(15) = 5.67$, $p<.001$, groups showed less dependency than the Dependent model. Experiment S2 therefore confirms the findings of experiment 2 & S1. An open-loop associative structure shows less dependency that a closed-loop structure, even when the number of elements within an event are controlled for.

Non-encoded pairs in the Open-loop condition

Finally, we analysed accuracy and dependency for pairs in the Open-loop condition that were not encoded. For example, if the participant encoded the location-person and location-object pairs for a specific event, we assessed accuracy for the non-encoded person-object pair. Dependency was assessed for the non-encoded pair relative to one of the other encoded pairs (e.g., cue person, retrieve object vs. retrieve location). Note, we were unable to perform this analysis for experiment 2 and S1 as participants were only tested on the encoded pairs.

Collapsing across all Cue-types and Retrieved-types, performance was 38%. This was significantly above chance (16.66% given the 6-alternative forced-choice), $t(15) = 4.63$, $p<.001$. However, we saw no evidence of dependency; dependency in the data (D = 0.56) did not differ from the Independent model (D = 0.55), $t(15) = .31$, $p=.76$, and was significantly less than the Dependent model (D = 0.60), $t(15) = 3.74$, $p<.01$. Thus, although participants can sometimes deduce the correct answer for a non-encoded pair, presumably via the encoded within-event associations, their performance for non-encoded pairs was not dependent on performance for the encoded pairs. We speculate that the noisy and inaccurate process that allows performance to be above chance for the unseen association (e.g. B-C) does utilize the encoded associations (e.g. A-B, A-C), but does so via a process that itself introduces too much variation across events to be sensitive to “dependency” (i.e., co-variation with performance across events in the much better recall of A-B and A-C).

Further analyses

Yule's Q

To complement the modelling approach used in the main analyses, we further computed Yule's Q for the data for each contingency table and each individual participant. Yule's Q is a measure of correlation within a 2x2 contingency table, varying from +1 (complete dependence), through zero (independence), to -1 (negative dependence) (S2). For a 2x2 contingency table, where a = correct/correct, b = correct/incorrect, c = incorrect/correct and d = incorrect/incorrect, Yule's Q = [ad-bc]/[ad+bc]. It has previously been used to assess dependency between recognition and recall, A-B vs. A-C associations (see also experiment S2), and symmetric vs. asymmetric associations within an A-B pair (see S3–7).

Yule's Q is highly sensitive to the presence of a zero in the 2x2 contingency table (which forces Q to be 1, -1, or undefined). Given the high proportion of tables containing one of more zeros across all experiments (mean: 37%), the main analyses used a proportional measure (i.e., the proportion of counts on the leading diagonal of the contingency table, see Experimental Procedures), comparing proportional dependency between the data and the models. To get around this problem (but compromising the interpretation of the outcome), we added 0.5 to all cells for every contingency table prior to calculating Yule's Q for the data.

Experiment 1 revealed mean Yule's Q values of 0.46 (SD=0.30) (a relatively strong positive correlation) for the Simultaneous condition and 0.50 (SD=0.31) for the Separated condition (collapsed across Analysis-type and Item-type). In experiment 2, mean Yule's Q for the Separated Closed-loop condition was 0.34 (SD=0.26), however for the Separated Open-

loop condition it was 0.02 (SD=0.25). Thus, for the Simultaneous and Separated Closed-loop condition Yule's Q was relatively high, ranging between 0.35 and 0.50, whereas for the Open-loop condition it was close to zero. This same pattern was seen for experiment S1 (see Supplemental Experimental Procedures), with Yule's Q in the Separated Closed-loop condition 0.33 (SD=0.27) and Separated Open-loop condition -0.09 (SD=0.19) and in experiment S2, with Yule's Q in the Separated Closed-loop condition 0.41 (SD=0.32) and Separated Open-loop condition 0.06 (SD=0.38).

Inter-experimental analyses

One difference between the results of experiment S2 and experiments 1 and 2 and S1 is that we saw significantly less dependency in the data than the Dependent model in the Separated Closed-loop condition (though dependency was still greater than the Independent model). It is unclear why this may have occurred, although a similar result was also seen in Horner & Burgess (S1) where all events were presented simultaneously. As such, less dependency than the Dependent model is unlikely to be a function of the separated encoding conditions in experiment S2. To further investigate this possible difference we conducted a 2x4 (Data vs. Dependent model x Experiment) mixed ANOVA, focussing on the difference in dependency in the data and Dependent model across experiments 1,2, S1 and S2. This revealed a significant difference between the data and Dependent model, $F(1, 55) = 15.43$, $p<.001$, $\eta_P^2=.22$. No main effect of Experiment was seen, $F(3, 55) = 2.38$, $p=.08$, $\eta_P^2=.12$ (though we note a trend), nor was there an interaction between the two factors, $F(3, 55) = 0.77$, $p=.52$, $\eta_P^2=.04$.

Thus, the difference between the data and Dependent model did not vary systematically across experiments. When pooling data across experiments, we see less

dependency in the data than Dependent model. However, note that our main conclusions are based on significant differences in dependency between the Closed-loop and Open-loop conditions (relative to the baseline of the Independent model), and do not rest on the absence of difference between dependency in the data and Dependent model in the Closed-loop condition. Note also that the Dependent model acts as an upper bound for the amount of dependency present in the data. Any source of (independent) noise will decrease dependency in the data, relative to the Dependent model.

Supplemental Experimental Procedures

Experiment 1

Participants – Twenty participants (10 female) gave informed consent to participate. The mean age was 25.0 (SD = 4.4). Four were excluded due to poor performance across all conditions (<30% accuracy), leaving 16 participants (1 left handed).

Materials – Stimuli were 36 locations (e.g., a swimming pool), famous personalities (e.g., David Cameron) and common objects (e.g., a bicycle; see S1). Four randomised sets of events (i.e., location-person-object triads) were created and counterbalanced across participants.

Procedure – Experiments consisted of single study and test phases. At study, participants were serially presented with all 36 events. Half the events were seen in single encoding trials containing three elements (Simultaneous Closed-loop condition), whereas the other half were seen as three overlapping pairs of elements across three separate encoding trials (Separated Closed-loop condition). The order of pair presentation for each "event" in the Separated Closed-loop condition was randomised such that, across participants, all possible pair orders will have been seen in roughly equal proportions. This resulted in 72 encoding trials, 18 triads and 54 pairs, all of which were presented in interleaved fashion (Fig 1).

At test, participants were presented with an element and had to choose the associated element from six alternatives. All 36 events were tested with every cue-test pair (e.g., cue: location, test: object), resulting in 6 cued-recognition trials per event. One of the test items was the element associated with the cue. The other 5 elements were of the same category (e.g., objects) but were associated with other elements, randomly selected from

Simultaneous Closed-loop and Separated Closed-loop events. Participants were required to respond as accurately as possible within 6secs with a key-press, and following this rated their confidence on a scale of 1-5.

Assessing Dependency – We created 2x2 contingency tables of each participant's performance for specific pairs of associations (e.g., A-B and A-C) across events. We assessed dependency for retrieving two elements (e.g., person and object) when cued by the remaining within-event element (e.g., location; 'A_BA_C' analyses), as well as for retrieving one element (e.g., location) when cued by its associated elements (e.g., person and object; 'B_AC_A' analyses). This resulted in six 2x2 tables per participant per condition, one for each element (item-type) and analysis-type.

Each contingency table shows how performance retrieving one association from an event (e.g., retrieving B cued by A) depends on performance retrieving another element from that event (e.g., retrieving C cued by A). For any given participant, the mean proportion of correct retrievals of B (over N events) when cued by A is denoted by P_{AB}. We then created tables for an Independent model for each participant, where the probability of correctly or incorrectly retrieving two associations is simply the product of the mean probability for each association (i.e., the proportion of events for which both B and C were correctly retrieved when cued by A would be $P_{AB}P_{AC}$), see Table 2.

The Dependent model modifies the Independent model by weighting performance by an "episodic factor" (E^i) that varies across events. This factor captures the extent to which the probability of correctly retrieving the elements comprising a specific event differs from the average probability across all events. For example, when retrieving B cued by A for event i:

$$E^i_{AB} = (T^i_{BA}+T^i_{BC}+T^i_{CA}+T^i_{CB})/(P_{BA}+P_{BC}+P_{CA}+P_{CB}) \quad (1)$$

where, T^i_{BA}=1 if the participant correctly retrieves A when cued by B (otherwise, T^i_{BA}=0), and similarly for T^i_{BC} etc. Note that the episodic factor, for retrievals cued by A, is estimated from retrievals not cued by A (i.e., excluding T^i_{AB}, T^i_{AC}, P_{AB}, P_{AC}). Note also that we have previously shown the Dependent model is not significantly affected by whether E^i is calculated with or without retrieval trials relating to the same cue element (e.g., calculating E^i_{AB} only using T^i_{BC} and T^i_{CB} compared to only using T^i_{AB} and T^i_{BA} (see S1). The probability of correctly retrieving an association from event *i* is weighted by the episodic factor for that event, i.e., P_{AB} becomes $\acute{P}_{AB}=E^i_{AB}P_{AB}$. The Dependent model also takes into account the level of guessing, so that E^i weights the probability of deliberate correct retrieval but not the probability of guessing correctly (which should be independent of other responses). So the Dependent model follows the Independent model, with P^i_{AB} (and similarly P^i_{AC}) replaced by:

$$\acute{P}^i_{AB} = E^i_{AB}(P_{AB}- P_G/c)+ P_G/c \quad (2)$$

where P_G is the proportion of guesses, of which P_G/c will be correct in c-way forced choice cued-recognition. P_G is estimated as $c/(c$-1) times the proportion of errors. See Table 2 and (S1). The Independent model corresponds to setting E^i = 1 across all events.

The two models estimate the contingency tables corresponding to independent or dependent retrieval of elements within an event, controlling for overall accuracy and the level of guessing. To compare the data with the models we calculated a dependency measure based on the proportion of events where the retrieval of two associations is either both correct or both incorrect, where 1 = full dependency and 0.5 = full independence. Note that this dependency measure is modulated by accuracy. As such, only comparisons

between dependency in the data and the models (which explicitly control for level of accuracy) are meaningful. After analysing dependency across the A_BA_C and B_AC_A analyses and across Item-types (see Supplemental Results), we averaged across these factors for comparisons between the data and the models. We also calculated Yule's Q measure of dependency (see S2–4) for the data for all experiments (see Supplemental Results).

Experiment 2

Experiment 2 was identical to experiment 1 with the following exceptions.

Participants – Sixteen participants (10 female) gave informed consent to participate. Their mean age was 23.4 (SD = 3.6). One was excluded due to poor performance across all conditions (<30% accuracy), leaving 15 participants (1 left-handed).

Materials – A further 36 animals (e.g., a monkey) were included in the stimulus set to create 4-element open-loop events (Fig 1).

Procedure – All "events" were presented as three separate pairs across three separate encoding trials (as in experiment 1 Separated Closed-loop condition; Fig 1). For the Separated Closed-loop condition, half the events were location-person-object (L-P-O) triads and the other half location-person-animal (L-P-A) triads. For the L-P-O triads, the presentation order of the pairs was P-O, L-O, L-P. For the L-P-A triads the order was L-A, P-A, L-P. For the Separated Open-loop condition half the events were presented in the order P-A, L-O, L-P; and half in the order L-O, P-A, L-P. The final pair presented for both conditions was always the location-person association. Whereas in experiment 1 we analysed dependency for all combinations of pairs, in experiment 2 we only analysed dependency for the location and person elements (relative to the other associated elements), as this controls for item

familiarity (each person and location being presented twice in each condition, once in a person-location association and once in association with another type of element). The test phase was identical to Experiment 1, all pairs presented during the study phase were tested in both directions (e.g., retrieve person cued by location, as well as retrieve location cued by person).

Analyses – We report accuracy for all four elements across the Separated Closed-loop and Separated Open-loop conditions. Note, however, that event numbers vary for the animal and object elements in the Separated Closed-loop condition as half the events were L-P-O triads and half were L-P-A triads. Dependency was assessed only for associations including location and person. For the Closed-loop condition, we assessed dependency between retrieval of the object (or animal) and person when cued by the location, and for retrieval of the object (or animal) and location when cued by the person (A_BA_C analyses). We also assessed dependency between retrieval of the location cued by the object (or animal) and by the person, and retrieval of the person when cued by the object (or animal) and by the location (B_AC_A analyses).

For the Open-loop condition, we assessed dependency between retrieval of the object and person when cued by the location, and retrieval of the animal and location when cued by the person (A_BA_C analyses). We also assessed dependency between retrieval of the location when cued by the object and by the person, and of the person when cued by the animal and location (B_AC_A analyses). This resulted in 4 dependency measures per participant. Independent and Dependent models were constructed for each contingency table, as in experiment 1. We again averaged across these conditions for comparisons of dependency between the data and models (see Supplemental Results for analyses of dependency for the

data across these conditions).

Experiment S1

Experiment S1 was identical to experiment 2 with the following exceptions.

Participants – 15 participants (12 female) gave informed consent to participate. Participants had a mean age of 22.5 (SD = 4.3). One was excluded due to poor performance across all conditions (<30% accuracy), leaving 14 participants. By self-report, all participants were right-handed.

Procedure – Experiment S1 was designed to rule out possible order effects at encoding in experiment 2. Whereas in experiment 2 the 1st and 2nd encoded pairs for each Open-loop "event" were unrelated (e.g., location-object then person-animal), the 2nd pair in experiment S1 always overlapped with the first encoded pair in both the Closed- and Open-loop conditions. Four orders were used for both conditions. In the Closed-loop condition, pairs were seen in the orders: (1) P-O, L-P, L-O (2) L-A, L-P, P-A (3) L-P, L-O, P-O and (4) L-P, L-A, P-A. In the Open-loop condition, pairs were seen in the orders: (1) P-A, L-P, L-O (2) L-O, L-P, P-A (3) L-P, L-O, P-A and (4) L-P, P-A, L-O.

Experiment S2

Experiment S2 was identical to experiment 2 with the following exceptions.

Participants - 34 participants (19 female) gave informed consent to participate. 17 participants were assigned to the Separated Closed-loop and 17 participants to the Separated Open-loop condition. Participants had a mean age of 24.1 (SD = 4.1). Three were excluded from the Closed-loop group and one from the Open-loop group due to poor

performance across all conditions (<30% accuracy), leaving 14 participants in the Closed-loop group and 16 in the Open-loop group. By self-report, four participants were left-handed, the remainder right-handed.

Procedure - Half of the participants saw all three pairs of an event at encoding (the Closed-loop condition), resulting in 108 trials at Study. The other half saw only two out of the possible three pairs of an event at encoding (the Open-loop condition), resulting in 72 trials at Study. Of the 36 events in the Open-loop group, 12 events presented the location-person and location-object pairs, 12 the location-person and object-person pairs and 12 the location-object and object-person pairs. As in experiment 1, the presentation order of pairs for each "event" was randomised. At test, all pairs were tested (including the non-encoded pairs in the Open-loop group).

Analyses - The main analyses of the Open-loop events were restricted to those pairs that were encoded. Thus, we assessed accuracy and dependency only for pairs that the participant had actually seen. For the dependency analysis this meant both associations for a particular contingency table (e.g., the A-to-B vs. A-to-C associations) had to have been seen by the participants at encoding. Analysis of the Closed-loop events was identical to experiment 1.

Supplemental References

S1. Horner AJ, Burgess N (2013) The associative structure of memory for multi-element events. *J Exp Psychol Gen* 142:1370–1383.

S2. Bishop YM, Fienberg SE, Holland PW (1975) *Discrete multivariate analysis: theory and practice* (MIT Press, Cambridge, Mass.).

S3. Kahana MJ (2000) in *Oxford handbook of human memory*, eds Tulving E, Craik FIM (Oxford Press, England), pp 323–284.

S4. Kahana MJ, Rizzuto DS, Schneider AR (2005) Theoretical correlations and measured correlations: relating recognition and recall in four distributed memory models. *J Exp Psychol Learn Mem Cogn* 31:933–953.

S5. Kahana MJ (2002) Associative symmetry and memory theory. *Mem Cognit* 30:823–840.

S6. Caplan JB, Glaholt MG, McIntosh AR (2006) Linking associative and serial list memory: Pairs versus triples. *J Exp Psychol Learn Mem Cogn* 32:1244–1265.

S7. Caplan JB, Rehani M, Andrews JC Associations compete directly in memory. *Q J Exp Psychol*.
