# Supplementary material for: Pattern Completion in Multielement Event Engrams
Source: Curr Biol. 2014 May 5;24(9):988–92. doi: 10.1016/j.cub.2014.03.012 (PMC4012134; doi:10.1016/j.cub.2014.03.012)
Supplement: Document S2. Article plus Supplemental Information [file mmc2.pdf]

Current Biology 24, 988–992, May 5, 2014 ©2014 The Authors http://dx.doi.org/10.1016/j.cub.2014.03.012

Report

Pattern Completion in Multielement Event Engrams

Aidan J. Horner[1,2,*] and Neil Burgess[1,2,*]
[1]UCL Institute of Cognitive Neuroscience, University College London, 17 Queen Square, London WC1N 3AR, UK
[2]UCL Institute of Neurology, University College London, Queen Square, London WC1 3BG, UK

Summary

Personally experienced events include multiple elements, such as locations, people, and objects. These events are thought to be stored in episodic memory as coherent representations [1] that allow the retrieval of all elements from a partial cue ("pattern completion" [2–6]). However, direct evidence for coherent multielement representations is lacking. Their presence would predict that retrieval of one element from an event should be dependent on retrieval of the other elements from that event. If we remember where we were, we should be more likely to remember who we met and what object they gave us. Here we provide evidence for this type of dependency in remembering three-element events. Dependency was seen when all three elements were encoded simultaneously, or when the three overlapping pairwise associations comprising an event were learned on separate trials. However, dependency was only seen in the separated encoding condition when all possible within-event associations were encoded. These results suggest that episodic memories are stored as coherent representations in which associations between all within-event elements allow retrieval via pattern completion. They also show that related experiences encountered at different times can be flexibly integrated into these coherent representations.

Results

Participants learned events composed of three or four elements (locations, people, and objects or animals) during a study phase (Figure 1 and Experimental Procedures). For example, for one event, they were presented with the words "kitchen," "Barack Obama," and "hammer" and required to imagine the three elements interacting. Using multielement events (as compared to simple pairwise associations) is critical to assess dependency. We can ask whether successful retrieval of one within-event association (e.g., retrieving location when cued with person) is dependent on retrieval of other associations from the same event (e.g., retrieving location when cued with object). During the test phase, each trial consisted of a cue (e.g., a location), with participants required to select the associated element (e.g., the person) among five other elements of the same type from different events. Each event was tested for all possible associations (e.g., location-person), in both directions, resulting in six retrieval trials per event.

*Correspondence: a.horner@ucl.ac.uk (A.J.H.), n.burgess@ucl.ac.uk (N.B.)
This is an open access article under the CC BY license (http://creativecommons.org/licenses/by/3.0/).

We created contingency tables showing the dependency in performance when retrieving different associations from the same event (following [7–10]), e.g., the 2 × 2 table for retrieving the object or the person when cued by the location. The dependency measure reflects the proportion of events in which both associations were retrieved correctly or both incorrectly. By comparing this dependency to Independent and Dependent models of retrieval, we assessed within-event dependency for each participant, controlling for their accuracy and level of guessing (see Table 2, Experimental Procedures, and Supplemental Information available online). The Independent model predicts the contingency table corresponding to unrelated retrievals of different associations from the same event. The Dependent model predicts the contingency table corresponding to dependent retrieval of all associations from the same event. The models provide lower and upper bounds to the expected level of dependency.

In experiment 1, half of the "events" were seen in single encoding trials containing all three elements (the Simultaneous condition; Figure 1A); the other half were seen as three overlapping pairs of elements across three separate encoding trials (the Separated Closed-Loop condition; Figures 1B and 1D). For example, in the Separated Closed-Loop condition, we presented the location and person on one trial, then the location and object, and finally the person and object (with each encoding trial separated by trials from other unrelated events). All 72 trials, corresponding to 18 events in each condition, were presented in interleaved order. Memory for each association (six per event) was then tested in 216 interleaved trials (six-alternative forced-choice).

Performance was good (76%; Table 1 and Supplemental Information). Contingency tables for the Simultaneous condition provided evidence for dependency (Figure 2A). Dependency exceeded the Independent model, $t(15) = 2.95$, $p < 0.01$, and did not differ from the Dependent model, $t(15) = 0.81$, $p = 0.43$ (see also [9]). The Separated Closed-Loop condition also showed greater dependency than the Independent model, $t(15) = 3.14$, $p < 0.01$, and did not differ from the Dependent model, $t(15) = 1.32$, $p = 0.21$. Importantly, the Simultaneous and Separated conditions showed similar dependency relative to their respective Independent models, $t(15) = 0.25$, $p = 0.81$.

Dependency comparable to the Dependent model was observed when the three elements of an "event" were presented simultaneously or in three separate pairwise encoding trials. These data suggest that episodic memories are stored as coherent representations and that related experiences encountered at different times can be integrated into these representations.

Experiment 2 aimed to replicate the finding of dependency in the Separated condition and to probe the conditions required for such dependency. In the Separated Closed-Loop condition, triads of the presented paired associates formed three-element events with an all-to-all or "closed-loop" associative structure (Figure 1D). Experiment 2 included triads of paired associates that formed four-element events (object-location-person-animal) with an open-loop associative structure (the Separated Open-Loop condition; Figure 1E). For example, participants would first encode

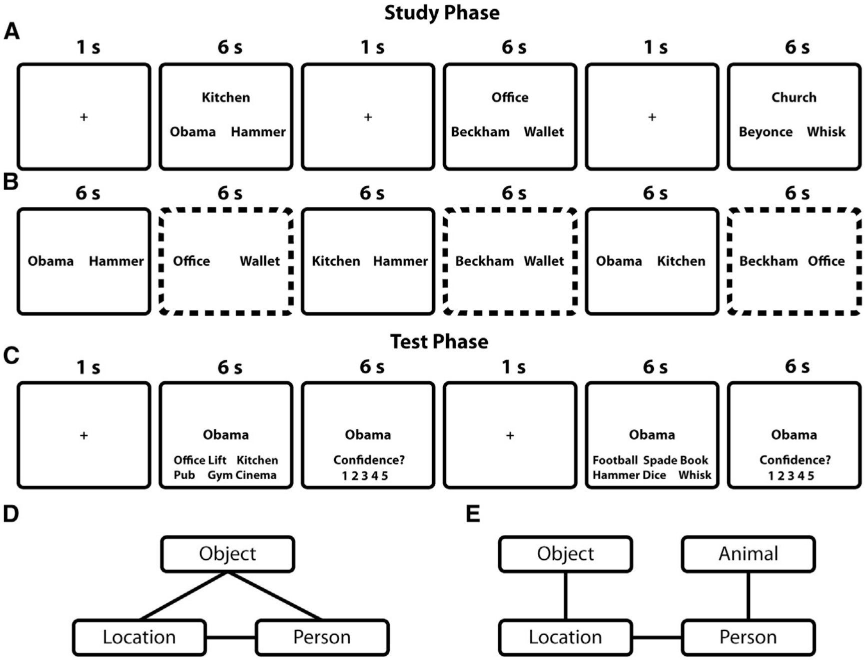

Figure 1. Trial Sequence for Study and Test Phases across Experiments

(A) Trial sequence and timing for the Simultaneous condition of experiment 1.

(B) Trial sequence (excluding 1 s fixation cross between encoding trials) for the Separated Closed-Loop and Separated Open-Loop conditions of experiments 1 and 2. Dotted lines are for illustrative purposes only (i.e., were not shown at encoding) to emphasize within-event pairs. Within-event pairs were not separated by a single intervening trial but had a mean of 36 intervening trials.

(C) Trial sequence of cued-recognition during the test phase of experiments 1 and 2. Within-event pairs were not tested consecutively but were separated by a mean of 36 intervening trials.

(D) Associative structure of the Simultaneous condition of experiment 1 and Separated Closed-Loop condition of experiments 1 and 2. (Note that half the Separated Closed-Loop events of experiment 2 were animal-location-person triads rather than object-location-person triads.)

(E) Associative structure of the Separated Open-Loop condition of experiment 2.

location-object, then person-animal, and finally location-person. The 108 paired associates for 18 events from each condition were presented in interleaved trials (nine Closed-Loop events contained object-location-person, and nine contained animal-location-person).

As in experiment 1, performance was good (65%; Table 1 and Supplemental Information), and we saw dependency for the Separated Closed-Loop condition: dependency exceeded the Independent model, $t(14) = 4.66$, $p < 0.001$, and did not differ from the Dependent model, $t(14) = 1.93$, $p = 0.07$ (though we note a trend; Figure 2B). By contrast, we saw no evidence for dependency in the Separated Open-Loop condition: dependency did not differ from the Independent model, $t(14) = 0.63$, $p = 0.54$, and was significantly less than the Dependent model, $t(14) = 4.78$, $p < 0.001$. Importantly, the Closed- and Open-Loop conditions differed in dependency relative to their respective Independent models, $t(14) = 3.48$, $p < 0.01$.

Dependency is seen when an event is presented across separate encoding trials (the Separated Closed-Loop condition). However, this dependency was only seen when all possible within-event pairs were encoded (i.e., the Separated Open-Loop condition did not show dependency). This lack of dependency in the Open-Loop condition was replicated, despite changing the order of the encoded pairs (experiment S1), and using paired associates from three-element events with an open-loop structure (where only two of the three possible associations were encoded; experiment S2). Thus, coherent event representations can be constructed across multiple encoding trials, but this depends on the associative structure presented, with a closed loop of three associations producing dependency, but not an open chain of three (or two) associations.

Discussion

Episodic memories are thought to be stored as coherent representations of the multiple elements comprising an event ("event engrams"). We found dependency in the retrieval of different elements of the same event, even when the event was formed from overlapping pairwise associations presented across separate trials, but only when all possible pairwise associations in the event were presented.

The associative structure of events has been investigated using partial cuing techniques [11, 12]; however, this approach does not address within-event dependency or variation across events. Dependency has also been assessed in memory for subordinate features (e.g., location on a screen and font size) of single elements (e.g., words) [13–15]. However, here we are interested in event memory, the binding of independently represented multimodal elements into coherent representations. Furthermore, these previous studies concerned "events" encoded on single trials and did not assess dependency for overlapping but independently encoded pairwise associations (i.e., our Separated conditions).

Dependency for events formed from simultaneously presented elements could reflect trial-by-trial modulation of attention (see [9]), since attention at encoding can modulate memory performance (e.g., [16–18]). However, we saw similar dependency for events formed from overlapping pairs of elements presented over different trials, ruling out an attentional explanation. These results also challenge models in which item information is associated via a time-varying context signal (e.g., [19, 20]), as its time-varying nature would cause independence in the Separated Closed-Loop condition. If a common "context" representation mediates within-event associations, it must predominantly comprise the within-event elements themselves. This forces us toward a mechanism in which within-event associations can be encoded independently but are retrieved in a dependent manner.

We suggest that dependency results from the associative structure of the "event." If all possible within-even pairs are encoded, a partial cue can cause retrieval of all within-event elements (regardless of whether they are being tested or not). This pattern completion process is thought to be a core function of the hippocampus [2–6], a region critical for

Table 1. Memory Performance across Experiments 1 and 2

		Retrieved Type			
	Cue Type	Location	Person	Object	Animal
Experiment 1					
Sim. Closed	Location	NA	0.80 (0.20)	0.72 (0.22)	NA
	Person	0.79 (0.23)	NA	0.76 (0.22)	NA
	Object	0.74 (0.23)	0.76 (0.20)	NA	NA
Sep. Closed	Location	NA	0.77 (0.18)	0.78 (0.20)	NA
	Person	0.77 (0.19)	NA	0.76 (0.26)	NA
	Object	0.77 (0.22)	0.79 (0.18)	NA	NA
Experiment 2					
Sep. Closed	Location	NA	0.64 (0.19)	0.68 (0.22)	0.80 (0.15)
	Person	0.60 (0.21)	NA	0.69 (0.19)	0.61 (0.14)
	Object	0.71 (0.18)	0.67 (0.22)	NA	NA
	Animal	0.70 (0.17)	0.64 (0.19)	NA	NA
Sep. Open	Location	NA	0.51 (0.22)	0.76 (0.20)	NA
	Person	0.51 (0.24)	NA	NA	0.58 (0.15)
	Object	0.75 (0.19)	NA	NA	NA
	Animal	NA	0.64 (0.18)	NA	NA

Proportion correct cued recognition (and SD) for each retrieved type (i.e., the element the participants were tested on; columns) and each cue type (i.e., the element the participants were cued with; rows) across the Simultaneous Closed-Loop (Sim. Closed) and Separated Closed-Loop (Sep. Closed) conditions of experiment 1 and Separated Closed-Loop and Separated Open-Loop (Sep. Open) conditions of experiment 2.

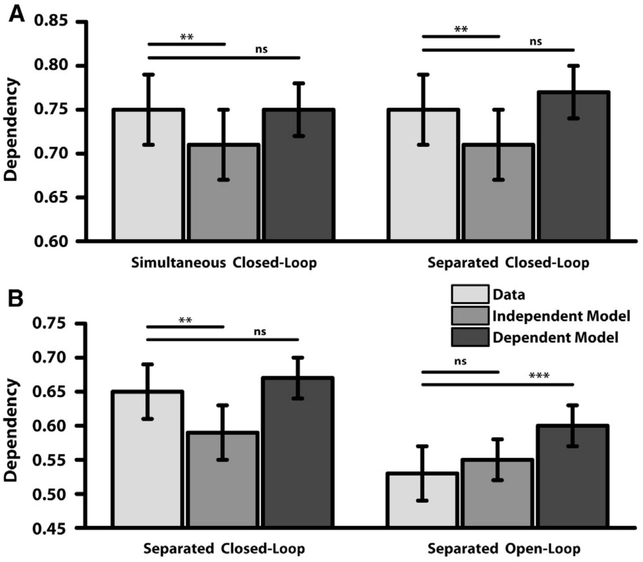

Figure 2. Dependency Analyses across Experiments 1 and 2

Dependency for the data, Independent model, and Dependent model across Simultaneous Closed-Loop and Separated Closed-Loop conditions of experiment 1 (A) and Separated Closed-Loop and Separated Open-Loop conditions of experiment 2 (B). Error bars represent ± 1 SE. ***$p < 0.001$; ** $p < 0.01$; ns, not significant.

episodic memory [21–23]. We suppose that hippocampal neurons selectively code for individual elements of any given event, consistent with "place cells" in rat [24] or human [25] hippocampus that represent specific locations and single neurons in the human hippocampus representing specific famous people [26].

In this view, it is the closed-loop structure of within-event associations that constitutes a coherent representation and allows pattern completion. This can be captured by simple autoassociative memory models (e.g., [2, 5, 6, 27]), in which reactivation of an individual element depends on the strengths of association between all elements within the event. In this case, retrieval performance on any one trial will reflect the strength of all within-event associations. The Dependent model captures this by assuming that performance on a retrieval question reflects the mean performance on the other questions regarding that event (the episodic factor *E*; see Experimental Procedures and Supplemental Information). Within this account, the lack of dependency in the Open-Loop condition reflects an absence of pattern completion: the only route for the cue to reactivate the target is via the cue-target association itself.

It is possible that pattern completion occurs at encoding (as well as at retrieval), allowing simultaneous encoding of all preceding within-event associations, which could introduce dependency in their strengths (see [28] for a related proposal). For example, when encoding the last pair (e.g., A-C), both the A-B and C-B associations could be retrieved, possibly leading to explicit imagery of all three elements. Note that this account still relies on the presence of pattern completion, albeit at encoding, and as such is constrained by the associative structure of the event: only occurring for closed-loop structures.

Our results have implications for how coherent event representations are formed from continuous experience. Features of the incoming stream of information can form contextual boundaries, segmenting our perception of the world into discrete events (e.g., [29, 30]) and influencing what information is bound within an event engram (e.g., [31, 32]). The presence of an "event boundary" can trigger the binding of all elements experienced in the preceding context, a process in which the hippocampus has been implicated (e.g., [33]). However, the presence of such an event boundary, demarcating a contiguous segment of time, may not be a necessary precondition for such binding to occur. Our results suggest that dependency can result from the associative structure of the related elements, rather than necessarily depending on their having been presented within the same context (cf. [19, 20]).

Although dependency was not seen for open-loop associative structures, presenting pairs A-B and A-C in experiment S2 led to above-chance performance for the nonencoded pairs (B-C), suggesting the presence of a weak association of nonencoded pairs, perhaps due to reactivation of A-B on presentation of A-C [28]. This association was presumably too weak to result in strong pattern completion of all three elements at retrieval, as dependency was not seen. Perhaps dependency for open-loop structures would be seen if nonencoded associations were sufficiently strengthened, by repetition or offline consolidation [34–37], potentially allowing for generalization across elements that have not been directly associated, a process that may also be mediated by the hippocampus [38–40].

The formation of integrated closed-loop structures over time might relate to the concept of "schema" [11, 41–43], consolidated memory structures that allow the integration of new related information. Our focus was on episodic memory and single presentations of memoranda, rather than long-term learning of statistical relationships over multiple presentations, which is the traditional focus of semantic learning and systems consolidation [2, 5, 44, 45] and in which "chunks" can be formed from higher-order relationships [46, 47]. Nonetheless, our "associative structures" may represent building blocks from which schema can be built. Although the exact relationships between traditionally defined "events," our

Table 2. The Independent and Dependent Models

	Retrieval of Element (B)	
Retrieval of Element (C)	Correct (P_{AB})	Incorrect ($1 - P_{AB}$)
Independent Model		
Correct (P_{AC})	$\sum_{i=1}^{N} P_{AB} P_{AC}$	$\sum_{i=1}^{N} P_{AC} (1 - P_{AB})$
Incorrect ($1 - P_{AC}$)	$\sum_{i=1}^{N} P_{AB} (1 - P_{AC})$	$\sum_{i=1}^{N} (1 - P_{AB})(1 - P_{AC})$
Dependent Model		
Correct (P_{AC})	$\sum_{i=1}^{N} \acute{P}^{i}_{AB} \acute{P}^{i}_{AC}$	$\sum_{i=1}^{N} \acute{P}^{i}_{AC} (1 - \acute{P}^{i}_{AB})$
Incorrect ($1 - P_{AC}$)	$\sum_{i=1}^{N} \acute{P}^{i}_{AB} (1 - \acute{P}^{i}_{AC})$	$\sum_{i=1}^{N} (1 - \acute{P}^{i}_{AB})(1 - \acute{P}^{i}_{AC})$

Contingency tables for the Independent and Dependent models, giving the frequency (over events) of the four combinations of correct or incorrect retrieval of elements B and C when cued by element A. The Dependent model replaces the probability of correctly recalling B when cued by A (across all events; P_{AB}) with $\acute{P}^{i}_{AB} = E^{i}_{AB}(P_{AB} - P_G/c) + P_G/c$, where the episodic factor E^{i}_{AB} reflects performance on event i relative to other events (based on retrievals other than B and C cued by A), P_G is the probability of guessing, and $c = 6$ is the number of choices in a test trial. P_{AC} is replaced similarly (see Supplemental Information for details). The Dependent model equates to the Independent model if the episodic factors are set to 1.

separately encoded "associative structures," and "schema" are currently unclear, our approach presents opportunities for bridging the fields of episodic memory and the segmentation, generalization, and consolidation of experience.

Conclusions

Theories of episodic memory propose the existence of coherent event representations, allowing retrieval of all event elements via pattern completion. Here we present evidence that such event engrams exist and are built from multiple overlapping associations that can be encoded independently. Performance in retrieving any within-event association is related to performance for retrieving other associations from the same event. We suggest this dependency results from a retrieval-related pattern completion process, requiring the presence of a closed-loop structure of associations between within-event elements. Our results shed light on how the episodic memory system can rapidly incorporate new information into associative structures, and how multielement events are retrieved through a process of pattern completion.

Experimental Procedures

Participants

All experiments were approved by the University College London Research Ethics Committee (NB/PWB/26102011a), and all participants gave informed consent (see Supplemental Information for participant details).

Materials

Stimuli were 36 locations (e.g., a swimming pool), famous personalities (e.g., David Cameron), common objects (e.g., a bicycle; see [9]), and animals (e.g., a dog).

Procedure

Experiments consisted of single study and test phases. At study, triads (for the Simultaneous condition) or pairs (for the Separated conditions) of elements were serially presented (Figure 1). Triads/pairs were presented for 6 s, as words, and participants were required to imagine the elements on the screen "interacting in a meaningful way as vividly as possible." Experiment 1 presented triads and pairs; experiment 2 presented only pairs (see Supplemental Information).

At test, participants were presented with an element and had to choose the associated element from six alternatives (Figure 1). All "events" were tested with every cue-test pair (e.g., cue: location, test: object), resulting in six cued-recognition trials per event. One of the test items was the element associated with the cue; the other five were elements of the same category (e.g., objects) randomly selected from other events (regardless of condition). Participants were required to respond as accurately as possible within 6 s with a key press and to rate their confidence on a scale of 1 to 5.

Assessing Dependency

We created 2 × 2 contingency tables of each participant's performance for specific pairs of associations across events, including tables for retrieving two elements (e.g., person and object) when cued by the remaining element (e.g., location; "A_BA_C" analyses) and for retrieving one element (e.g., location) when cued by its associated elements (e.g., person and object; "B_AC_A" analyses). This resulted in six 2 × 2 tables per participant per condition in experiments 1 and S2, one for each element (item type) and analysis type (A_BA_C or B_AC_A), and four tables per participant per condition in experiments 2 and S1 (testing the four pairs of associations common to both Open- and Closed-Loop conditions; see Supplemental Information).

To assess dependency, we took the proportion of events in which both associations were either correctly or incorrectly retrieved and averaged this measure across the contingency tables for a given condition. We also calculated Yule's Q measure of dependency (see [8, 10, 48]) for the data for all experiments (see Supplemental Information).

For each contingency table, we created predicted tables corresponding to "Independent" and "Dependent" models of retrieval (see Table 2, Supplemental Information, and [9] for details). The Independent model predicts the dependency corresponding to the participant's mean level of performance for the two associations across events. In the Dependent model, the predicted retrieval performance for a given question is adjusted by the mean performance over other questions for that event (the episodic factor E). It predicts the maximal level of dependency, given the participant's mean level of performance for the two associations, their overall level of guessing, and the amount of variance in their overall performance across events. The Independent and Dependent models serve as theoretical lower and upper bounds for comparison to the level of dependency in the data, for each participant in each condition.

Supplemental Information

Supplemental Information includes Supplemental Results, one figure, and Supplemental Experimental Procedures and can be found with this article online at http://dx.doi.org/10.1016/j.cub.2014.03.012.

Acknowledgments

We thank Dharshan Kumaran and Peter Dayan for helpful comments on an earlier version of this manuscript. This work was funded by the Medical Research Council UK and the Wellcome Trust.

Received: November 21, 2013
Revised: January 29, 2014
Accepted: March 6, 2014
Published: April 17, 2014

References

1. Tulving, E. (1983). Elements of Episodic Memory (Oxford: Clarendon Press).
2. McClelland, J.L., McNaughton, B.L., and O'Reilly, R.C. (1995). Why there are complementary learning systems in the hippocampus and neocortex: insights from the successes and failures of connectionist models of learning and memory. Psychol. Rev. *102*, 419–457.
3. Wills, T.J., Lever, C., Cacucci, F., Burgess, N., and O'Keefe, J. (2005). Attractor dynamics in the hippocampal representation of the local environment. Science *308*, 873–876.
4. Nakazawa, K., Quirk, M.C., Chitwood, R.A., Watanabe, M., Yeckel, M.F., Sun, L.D., Kato, A., Carr, C.A., Johnston, D., Wilson, M.A., and Tonegawa, S. (2002). Requirement for hippocampal CA3 NMDA receptors in associative memory recall. Science *297*, 211–218.
5. Marr, D. (1971). Simple memory: a theory for archicortex. Philos. Trans. R. Soc. Lond. B Biol. Sci. *262*, 23–81.
6. Gardner-Medwin, A.R. (1976). The recall of events through the learning of associations between their parts. Proc. R. Soc. Lond. B Biol. Sci. *194*, 375–402.

7. Tulving, E., and Watkins, M.J. (1975). Structure of memory traces. Psychol. Rev. *82*, 261–275.
8. Kahana, M.J. (2000). Contingency analyses of memory. In Oxford Handbook of Human Memory, E. Tulving and F.I.M. Craik, eds. (Oxford: Oxford University Press), pp. 59–72.
9. Horner, A.J., and Burgess, N. (2013). The associative structure of memory for multi-element events. J. Exp. Psychol. Gen. *142*, 1370–1383.
10. Kahana, M.J., Rizzuto, D.S., and Schneider, A.R. (2005). Theoretical correlations and measured correlations: relating recognition and recall in four distributed memory models. J. Exp. Psychol. Learn. Mem. Cogn. *31*, 933–953.
11. Ross, B.H., and Bower, G.H. (1981). Comparisons of models of associative recall. Mem. Cognit. *9*, 1–16.
12. Jones, G.V. (1976). A fragmentation hypothesis of memory: cued recall of pictures and of sequential position. J. Exp. Psychol. Gen. *105*, 277–293.
13. Meiser, T., and Bröder, A. (2002). Memory for multidimensional source information. J. Exp. Psychol. Learn. Mem. Cogn. *28*, 116–137.
14. Starns, J.J., and Hicks, J.L. (2005). Source dimensions are retrieved independently in multidimensional monitoring tasks. J. Exp. Psychol. Learn. Mem. Cogn. *31*, 1213–1220.
15. Meiser, T., Sattler, C., and Weisser, K. (2008). Binding of multidimensional context information as a distinctive characteristic of remember judgments. J. Exp. Psychol. Learn. Mem. Cogn. *34*, 32–49.
16. Jacoby, L.L., Toth, J.P., and Yonelinas, A.P. (1993). Separating conscious and unconscious influences of memory: measuring recollection. J. Exp. Psychol. Gen. *122*, 139–154.
17. Eich, E. (1984). Memory for unattended events: remembering with and without awareness. Mem. Cognit. *12*, 105–111.
18. Bentin, S., Moscovitch, M., and Nirhod, O. (1998). Levels of processing and selective attention effects on encoding in memory. Acta Psychol. (Amst.) *98*, 311–341.
19. Estes, W.K. (1955). Statistical theory of spontaneous recovery and regression. Psychol. Rev. *62*, 145–154.
20. Howard, M.W., and Kahana, M.J. (2002). A distributed representation of temporal context. J. Math. Psychol. *46*, 269–299.
21. Scoville, W.B., and Milner, B. (1957). Loss of recent memory after bilateral hippocampal lesions. J. Neurol. Neurosurg. Psychiatry *20*, 11–21.
22. Squire, L.R., and Zola-Morgan, S. (1991). The medial temporal lobe memory system. Science *253*, 1380–1386.
23. Kinsbourne, M., and Wood, F. (1975). Short-term memory processes and the amnesic syndrome. In Short-Term Memory, D. Deutsch and A.J. Deutsch, eds. (New York: Academic Press), pp. 257–291.
24. O'Keefe, J., and Dostrovsky, J. (1971). The hippocampus as a spatial map. Preliminary evidence from unit activity in the freely-moving rat. Brain Res. *34*, 171–175.
25. Ekstrom, A.D., Kahana, M.J., Caplan, J.B., Fields, T.A., Isham, E.A., Newman, E.L., and Fried, I. (2003). Cellular networks underlying human spatial navigation. Nature *425*, 184–188.
26. Quiroga, R.Q., Reddy, L., Kreiman, G., Koch, C., and Fried, I. (2005). Invariant visual representation by single neurons in the human brain. Nature *435*, 1102–1107.
27. Hopfield, J.J. (1982). Neural networks and physical systems with emergent collective computational abilities. Proc. Natl. Acad. Sci. USA *79*, 2554–2558.
28. Zeithamova, D., Dominick, A.L., and Preston, A.R. (2012). Hippocampal and ventral medial prefrontal activation during retrieval-mediated learning supports novel inference. Neuron *75*, 168–179.
29. Speer, N.K., Zacks, J.M., and Reynolds, J.R. (2007). Human brain activity time-locked to narrative event boundaries. Psychol. Sci. *18*, 449–455.
30. Zacks, J.M., Braver, T.S., Sheridan, M.A., Donaldson, D.I., Snyder, A.Z., Ollinger, J.M., Buckner, R.L., and Raichle, M.E. (2001). Human brain activity time-locked to perceptual event boundaries. Nat. Neurosci. *4*, 651–655.
31. Ezzyat, Y., and Davachi, L. (2011). What constitutes an episode in episodic memory? Psychol. Sci. *22*, 243–252.
32. DuBrow, S., and Davachi, L. (2013). The influence of context boundaries on memory for the sequential order of events. J. Exp. Psychol. Gen. *142*, 1277–1286.
33. Ben-Yakov, A., and Dudai, Y. (2011). Constructing realistic engrams: poststimulus activity of hippocampus and dorsal striatum predicts subsequent episodic memory. J. Neurosci. *31*, 9032–9042.
34. Wagner, U., Gais, S., Haider, H., Verleger, R., and Born, J. (2004). Sleep inspires insight. Nature *427*, 352–355.
35. Stickgold, R., and Walker, M.P. (2013). Sleep-dependent memory triage: evolving generalization through selective processing. Nat. Neurosci. *16*, 139–145.
36. Durrant, S.J., Cairney, S.A., and Lewis, P.A. (2013). Overnight consolidation aids the transfer of statistical knowledge from the medial temporal lobe to the striatum. Cereb. Cortex *23*, 2467–2478.
37. Dumay, N., and Gaskell, M.G. (2007). Sleep-associated changes in the mental representation of spoken words. Psychol. Sci. *18*, 35–39.
38. Eichenbaum, H. (2004). Hippocampus: cognitive processes and neural representations that underlie declarative memory. Neuron *44*, 109–120.
39. Kumaran, D., and McClelland, J.L. (2012). Generalization through the recurrent interaction of episodic memories: a model of the hippocampal system. Psychol. Rev. *119*, 573–616.
40. Shohamy, D., and Wagner, A.D. (2008). Integrating memories in the human brain: hippocampal-midbrain encoding of overlapping events. Neuron *60*, 378–389.
41. Bartlett, F.C. (1932). Remembering: A Study in Experimental and Social Psychology (Cambridge: Cambridge University Press).
42. van Kesteren, M.T.R., Fernández, G., Norris, D.G., and Hermans, E.J. (2010). Persistent schema-dependent hippocampal-neocortical connectivity during memory encoding and postencoding rest in humans. Proc. Natl. Acad. Sci. USA *107*, 7550–7555.
43. Tse, D., Langston, R.F., Kakeyama, M., Bethus, I., Spooner, P.A., Wood, E.R., Witter, M.P., and Morris, R.G.M. (2007). Schemas and memory consolidation. Science *316*, 76–82.
44. Alvarez, P., and Squire, L.R. (1994). Memory consolidation and the medial temporal lobe: a simple network model. Proc. Natl. Acad. Sci. USA *91*, 7041–7045.
45. Nadel, L., and Moscovitch, M. (1997). Memory consolidation, retrograde amnesia and the hippocampal complex. Curr. Opin. Neurobiol. *7*, 217–227.
46. Orbán, G., Fiser, J., Aslin, R.N., and Lengyel, M. (2008). Bayesian learning of visual chunks by human observers. Proc. Natl. Acad. Sci. USA *105*, 2745–2750.
47. Schapiro, A.C., Rogers, T.T., Cordova, N.I., Turk-Browne, N.B., and Botvinick, M.M. (2013). Neural representations of events arise from temporal community structure. Nat. Neurosci. *16*, 486–492.
48. Bishop, Y.M., Fienberg, S.E., and Holland, P.W. (1975). Discrete Multivariate Analysis: Theory and Practice (Cambridge: MIT Press).

Current Biology, Volume *24*

Supplemental Information

Pattern Completion
in Multielement Event Engrams

Aidan J. Horner and Neil Burgess

Supplemental Information

Supplemental Results

Further analyses of Experiments 1 & 2

Cued-recognition performance

Experiment 1

A 2x3 (Simultaneous vs. Separated x Cue-type) within-subjects ANOVA of retrieval performance, collapsed across Retrieved-type, failed to reveal any significant effects, *F*'s<1.07, p's>.34, η_P^{2}'s<.07. A similar 2x3 (Simultaneous vs. Separated x Retrieved-type) ANOVA, collapsed across Cue-type, also failed to reveal any significant effects, *F*'s<1.35, p's>.27, η_P^{2}'s<.08. Thus, no differences in performance were seen across the three item types (location, people and objects), either as the cue-type or retrieved-type. Further, no difference in performance was seen between the Simultaneous and Separated conditions.

Experiment 2

A 2x4 (Closed-loop vs. Open-loop x Cue-type) within-subjects ANOVA of retrieval performance, collapsed across Retrieved-type, revealed a Closed-loop vs. Open-loop x Cue-type interaction, $F(1.6, 22.1) = 4.58$, $p<.05$, $\eta_P^2=.25$, and a main effect of Cue-type, $F(2.1, 29.5) = 6.56$, $p<.001$, $\eta_P^2=.32$. Despite a trend, no significant difference was seen between Closed-loop vs. Open-loop, $F(1, 14) = 3.86$, $p=.07$, $\eta_P^2=.22$. The interaction was characterised by an effect of Cue-type in the Open-loop condition, $F(2.0, 27.4) = 9.03$, $p<.01$, $\eta_P^2=.39$, with better performance when cued by an object relative to the other three elements, but no Cue-type effect in the Closed-loop condition, $F(1.9, 26.2) = 1.67$, $p=.21$, $\eta_P^2=.11$.

A similar 2x4 (Closed-loop vs. Open-loop x Retrieved-type) ANOVA, collapsed across Cue-type, revealed a similar Closed-loop vs. Open-loop x Cue-type interaction, $F(3.0, 41.38) = 12.24$, $p<.001$, $\eta_P^2=.47$, as well as main effects of Retrieved-type, $F(2.1, 30.1) = 5.68$, $p<.01$, $\eta_P^2=.29$, and of Closed-loop vs. Open-loop, $F(1, 14) = 6.34$, $p<.05$, $\eta_P^2=.31$. Again, the interaction was characterised by an effect of Retrieved-type in the Open-loop condition, $F(2.5, 35.3) = 11.04$, $p<.001$, $\eta_P^2=.44$, with better performance when retrieving the object relative to the other three elements, but no Retrieved-type effect in the Closed-loop condition, $F(1.8, 24.9) = 1.77$, $p=.19$, $\eta_P^2=.11$. To summarise, performance was better in the Closed-loop than Open-loop condition, though this only reached significance in the analysis across Retrieved-type, and performance was more variable across the four elements in the Open-loop than Closed-loop condition.

The differences in memory performance across the four item-types (locations, people, objects and animals) in the Open-loop but not Closed-loop condition suggest an underlying difference in memory strength across item types, with better memory for objects. However, when dependency is seen (i.e., in the Closed-loop condition), accuracy becomes more similar across item types. This finding is consistent with the presence of pattern completion in the Closed-loop condition, in which performance reflects all within-event associations rather than just the association between the specific elements tested.

Analysis of dependency across Analysis-type and Item-type

Experiment 1

A 2x2x3 (Simultaneous vs. Separated x Analysis-type x Item-type) ANOVA on dependency of the observed data failed to reveal any main effects or an interaction (F’s<2.23, p’s>.15,

η_P^2's<.13). Analysis-type refers to 'A_BA_C' versus 'B_AC_A' analyses (see Experimental Procedures). Item-type refers to the type of the common element in the dependency analysis (i.e., the element referred to as A in the above description of analysis-type).

Experiment 2

A 2x2x2 (Closed-loop vs. Open-loop x Analysis-type x Item-type) ANOVA on dependency of the observed data revealed a main effect of Closed-loop vs. Open-loop, $F(1, 14) = 12.87$, $p<.01$, $\eta_P^2=.48$, reflecting the difference in dependency between the two conditions (but potentially also reflecting accuracy differences, which are controlled for by the comparisons to Independent and Dependent models in the main text). No further main effects or interactions reached significance, F's<1.7, p's>.22, η_P^2's<.11.

Table S1, related to Figure 2. Mean dependency (D) (and standard deviation) for the A_BA_C and B_AC_A analyses for the observed data across the Simultaneous Closed-loop (Sim. Closed) and Separated Closed-loop (Sep. Closed) conditions of Experiment 1 and Separated Closed-loop (Sep. Closed) and Separated Open-loop (Sep. Open) conditions of Experiment 2.

Analysis:	A_BA_C			B_AC_A		
Item-type:	Location	Person	Object	Location	Person	Object
Experiment 1						
Sim. Closed	.76 (.14)	.77 (.17)	.74 (.16)	.76 (.17)	.71 (.21)	.74 (.15)
Sep. Closed	.79 (.17)	.77 (.15)	.73 (.19)	.75 (.18)	.74 (.18)	.75 (.18)
Experiment 2						
Sep. Closed	.69 (.16)	.61 (.18)	n/a	.63 (.13)	.68 (.15)	n/a
Sep. Open	.55 (.17)	.54 (.17)	n/a	.53 (.20)	.53 (.13)	n/a

Analysis of encoding order in the Separated Closed-loop condition

In Horner & Burgess (S1), we raised the possibility that dependency could change across the 6 retrieval trials per event. Interestingly, although we saw an increase in accuracy from retrieval trial 1-6, we saw no evidence for any increase in dependency when pairing the 1st and 2nd, 3rd and 4th and 5th and 6th retrieval trials for each event. In fact, the analyses raised the possibility of a small decrease in dependency, once the overall increase in accuracy was taken into account. In previous studies, an 'event' was presented in a single trial with all elements shown on the screen at the same time (equivalent to the Simultaneous condition of experiment 1). Given that we introduced a 'Separated' condition for the current studies, we can ask a similar question at encoding. Does dependency differ as a function of when each pairwise association was encoded? To do this, we paired up retrieval trials for the 1st vs. 2nd encoded pair, 2nd vs. 3rd encoded pair and 1st vs. 3rd encoded pair of each event, and conducted the same dependency analyses as in the main text. Note, this analysis was only performed on the Separated Closed-loop conditions of experiments 1 and 2 (as these were the Separated conditions that showed evidence for dependency).

Experiment 1

A one-way ANOVA (1st vs. 2nd vs. 3rd encoded pair) analysing cued-recognition performance showed a significant main effect, $F(1.7, 25.6) = 3.63$, $p<.05$, $\eta_P^2=.20$, revealing greater performance for 1st (81%), than 2nd (76%) and 3rd (75%) encoded pairs. However, dependency does not appear to be affected by encoding order. A one-way ANOVA comparing the raw dependency measure for 1st vs. 2nd, 2nd vs. 3rd and 1st vs. 3rd encoded pairs was not significant, $F(1.8, 27.5) = .93$, $p=.41$, $\eta_P^2=.06$. A similar ANOVA on the difference between dependency in the data and the Independent model (i.e., controlling for

accuracy differences) for the same 3 pairings was also not significant, $F(1.7, 26.8) = .84$, $p=.43$, $\eta_P^2=.05$. Thus, we could find no evidence for differences in dependency as a function of the order of encoded pairs.

Experiment 2

A one-way ANOVA (1st vs. 2nd vs. 3rd encoded pair) analysing cued-recognition performance showed a significant main effect, $F(1.8, 25.7) = 6.91$, $p<.01$, $\eta_P^2=.33$, revealing greater performance for 1st (72%), than 2nd (66%) and 3rd (62%) encoded pairs. Again, dependency does not appear to be affected by encoding order. A one-way ANOVA comparing the raw dependency measure for 1st vs. 2nd, 2nd vs. 3rd and 1st vs. 3rd encoded pairs was not significant, $F(1.5, 21.5) = 2.05$, $p=.16$, $\eta_P^2=.13$. A similar ANOVA on the difference between dependency in the data and the Independent model (i.e., controlling for accuracy differences) for the same 3 pairings did reach significance, $F(1.9, 27.3) = 3.79$, $p<.05$, $\eta_P^2=.21$, with less dependency for the 1st vs. 2nd pair (dependency for Data – Independent model = 0.01) than 2nd vs. 3rd (0.06) or 1st vs. 3rd (0.04).

Although this difference was unexpected, a full 2x3 mixed ANOVA across experiments 1 and 2, comparing the difference between the data and Independent model across the 3 pairings failed to reveal a main effect of encoding pair, $F(1.9, 55.3) = 1.35$, $p=.27$, $\eta_P^2=.05$, with no interaction between this factor and Experiment, $F(1.9, 27.3) = 2.78$, $p=.07$, $\eta_P^2=.08$ (though we note a trend). Thus, across experiments we could not find consistent evidence for differences in dependency as a function of the order of encoded pairs.

Analysis of reaction times

Though not central to our main hypotheses, it is conceivable that differences in reaction times (RTs) between our conditions of interest may have contributed to differences in dependency. For example, perhaps longer RTs in one condition might allow participants to explicitly retrieve all associated elements of an event, leading to increases in dependency for that condition (i.e., such a finding might help to interpret our observations of dependency). In experiment 1, no difference was seen between the Simultaneous (mean: 3270msecs) and Separated Closed-loop (3233msecs) conditions, $t(15) = .53$, $p=.60$. In experiment 2, no difference was seen between the Separated Closed-loop (3550msecs) and Separated Open-loop (3668msecs) conditions, $t(14) = 1.46$, $p=.17$. Thus, we could find no evidence for RT differences at retrieval between our main experimental conditions.

Supplemental Experiments S1 & S2

Experiment S1 – Closed- vs. Open-loop 4-item events

The Open-loop condition of experiment 2 presented the paired associates for each event in a specific order. For example, if the location-object pair was encoded first, the 2nd pair would have been person-animal and the last pair was always location-person (see Supplemental Experimental Procedures). Thus, the 2nd encoded pair never overlapped with the 1st encoded pair. This was not the case in the Closed-loop condition, where the 2nd encoded pair by necessity had to overlap with the 1st encoded pair. Experiment S1 controlled for this possible encoding order issue. Regardless of the 1st encoded pair, the 2nd encoded pair in the Open-loop condition always overlapped with the 1st pair (see

Supplemental Experimental Procedures). For example, if participants first learned location-object, the next encoded pair would be location-person and then person-animal. Thus, any lack of dependency in the Separated Open-loop condition of experiment S1 could not result from the fact that the first two learned pairs for any 'event' were completely unrelated (and only subsequently connected by the final encoded pair).

Cued-recognition performance

Performance was well above chance for both the Closed-loop (61%) and Open-loop (62%) conditions. A 2x4 (Closed-loop vs. Open-loop x Cue-type) within-subjects ANOVA on retrieval performance, collapsed across Retrieved-type, revealed a Closed-loop vs. Open-loop x Cue-type interaction, $F(2.6, 34.2) = 7.43$, $p<.01$, $\eta_P^2=.36$, as well as a main effect of Cue-type, $F(2.7, 35.5) = 3.49$, $p<.05$, $\eta_P^2=.21$. No main effect of Closed- vs. Open-loop was seen, $F(1, 13) = .23$, $p=.64$, $\eta_P^2=.02$. The interaction was characterised by a larger effect of Cue-type in the Open-loop condition, $F(2.6, 33.5) = 9.21$, $p<.001$, $\eta_P^2=.42$, relative to the Closed-loop condition, $F(2.8, 36.1) = 4.42$, $p<.05$, $\eta_P^2=.25$, with better performance when cued by an object relative to the other three elements in the Open-loop condition. A similar 2x4 (2x4 (Closed-loop vs. Open-loop x Retrieved-type) ANOVA, collapsed across Cue-type, failed to reveal any significant effects or interactions, *F*'s<2.2, p's>.13. As in experiment 2, performance was more variable across item-type in the Open-loop than Closed-loop condition (albeit only for the Cue-type analysis in experiment S1).

Dependency analysis

A 2x2x2 (Closed-loop vs. Open-loop x Analysis-type x Item-type) ANOVA on dependency of the observed data revealed a main effect of Closed-loop vs. Open-loop, $F(1, 13) = 18.62$,

$p<.001$, $\eta_P^2=.59$ (as in Experiment 2). Apart from a trend towards a Closed- vs. Open-loop x Item-type interaction, $F(1, 13) = 3.49$, $p=.09$, $\eta_P^2=.21$, no further main effects or interactions were seen, F's<1.3, p's>.26. As in experiments 1 & 2, we collapsed across Analysis-type and Item-type for comparisons between the data and the models.

Dependency for the data, Independent model and Dependent model for Closed-loop and Open-loop events is shown in Figure S1. For the Closed-loop events, dependency was greater than the Independent model, $t(13) = 2.85$, $p<.05$, and did not differ from the Dependent model, $t(13) = 1.41$, $p=.18$. For the Open-loop events, dependency did not differ from the Independent model, $t(13) = 1.89$, $p=.08$ (though we note a trend in the opposite direction, i.e., less dependency in the data than the Independent model), but was less than the Dependent model, $t(13) = 8.35$, $p<.001$. Importantly, the difference in dependency between the data and Independent model (Data – Independent model) was greater in the Closed-loop than Open-loop condition, $t(13) = 3.37$, $p<.01$. Experiment S1 therefore replicates the results of experiment 2. An open-loop associative structure shows less dependency than a closed-loop structure, even when we change the encoding order to ensure all 2nd encoded pairs overlap with 1st encoded pairs regardless of condition.

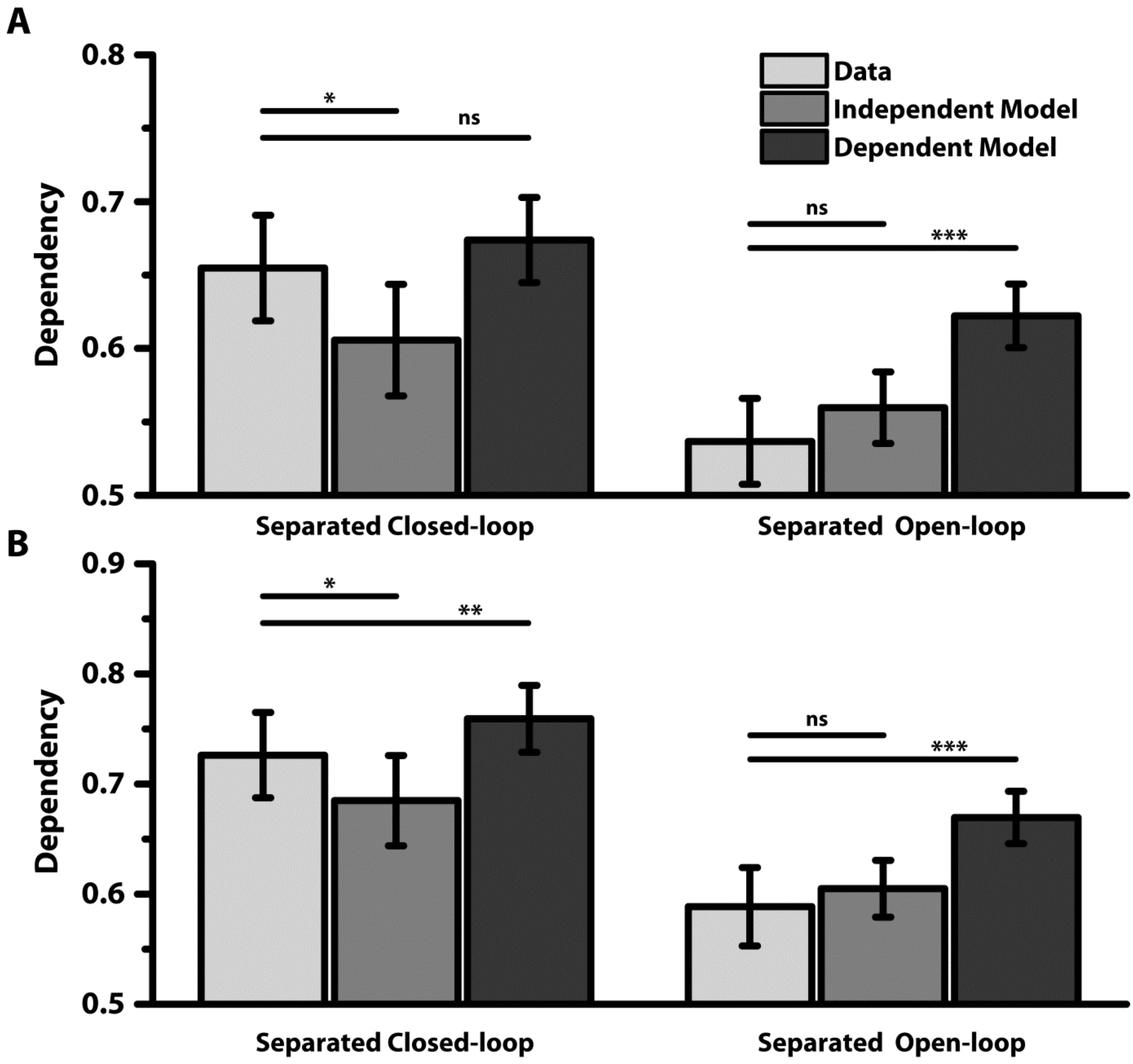

Figure S1, related to Figure 2. Dependency for the data, Independent model and Dependent model across the Separated Closed-loop and Separated Open-loop conditions of (a) experiment S1 and (b) experiment S2. Error bars represent +/- 1 standard error; *** $p<.001$; ** $p<.01$; * $p<.05$; ns = not significant.

Experiment S2 – Closed- vs. Open-loop 3-item events

The Open-loop condition of experiments 2 & S1 included a fourth element in the associative structure of the event engram. This was done to equate the number of associations within the engram, as well as familiarity with the location and person elements of the events, between the Open-loop and Closed-loop conditions. However, it is possible that it was the

fourth element, as opposed to the open-loop structure, of the event engram that resulted in the decrease in dependency seen in experiment 2 and S1. To rule out this possibility, we ran a fourth experiment where participants were only presented with 3-element events (location-person-object triads).

In a between-subject manipulation, half the participants were presented with all three possible pairs during encoding (across separate encoding trials; the Separated Closed-loop condition) and half the participants were presented with only two out of the three possible pairs (the Separated Open-loop condition). For example, they might be shown the location-person and person-object pair, but not the location-object pair, at encoding (see Supplemental Experimental Procedures). Therefore, the Open-loop condition of experiment S2 controls for the number of elements within an 'event', but not the number of associations, whereas the Open-loop condition of experiment 2 and S1 controlled for the number of associations, but not the number of elements. If the decrease in dependency in experiment 2 and S1 was due to the open-loop associative structure of the event engram (as opposed to the number of elements within an event), we should see a similar decrease in dependency when only two out of the three possible pairs of a triad are presented.

Cued-recognition performance

Performance was well above chance for both the Closed-loop (75%) and Open-loop (66%) conditions. A 2x3 (Closed-loop vs. Open-loop x Cue-type) mixed ANOVA failed to reveal any significant effects on performance, *F*'s<1.70, p's>.20, η_P^2's<.06. A similar ANOVA across Retrieved-type showed a trend for a main effect of Retrieved-type, $F(1.9, 53.2) = 3.16$, $p=.05$, $\eta_P^2=.10$. This effect appeared to be driven by greater accuracy when retrieving the Person relative to Locations and Objects. No further significant effects were seen, *F*'s<1.63,

p's>.21, η_P^{2}'s<.06. Thus, no difference was seen in accuracy between the Closed-loop and Open-loop group.

Dependency analysis

A 2x2x3 (Closed-loop vs. Open-loop x Analysis-type x Item-type) mixed ANOVA on dependency of the observed data revealed a main effect of Closed-loop vs. Open-loop, $F(1, 28) = 6.08$, $p<.05$, $\eta_P^2=.18$, with greater dependency in the Closed-loop than Open-loop condition. Apart from a Closed-loop vs. Open-loop x Item-type interaction, $F(1.8, 49.7) = 4.79$, $p<.05$, $\eta_P^2=.15$, no further significant effects were seen, F's<2.70, p's>.08, η_P^{2}'s<.09. As in experiments 1 & 2, we collapsed across Analysis-type and Item-type for comparisons between the data and the models.

Dependency for the data, Independent model and Dependent model for Closed-loop and Open-loop events is shown in Figure S1. For the Closed-loop events, dependency was greater than the Independent model, $t(13) = 2.76$, $p<.05$, replicating the findings of experiments 1,2 & S1. As in experiment 2 & S1, we saw no difference between the data and Independent model for the Open-loop events, $t(15) = .94$, $p=.36$. Importantly, the difference between the data and Independent model for the Closed-loop group was greater than for the Open-loop group, $t(28) = 2.41$, $p<.05$. Finally, both the Closed-loop, $t(13) = 3.29$, $p<.01$, and Open-loop, $t(15) = 5.67$, $p<.001$, groups showed less dependency than the Dependent model. Experiment S2 therefore confirms the findings of experiment 2 & S1. An open-loop associative structure shows less dependency that a closed-loop structure, even when the number of elements within an event are controlled for.

Non-encoded pairs in the Open-loop condition

Finally, we analysed accuracy and dependency for pairs in the Open-loop condition that were not encoded. For example, if the participant encoded the location-person and location-object pairs for a specific event, we assessed accuracy for the non-encoded person-object pair. Dependency was assessed for the non-encoded pair relative to one of the other encoded pairs (e.g., cue person, retrieve object vs. retrieve location). Note, we were unable to perform this analysis for experiment 2 and S1 as participants were only tested on the encoded pairs.

Collapsing across all Cue-types and Retrieved-types, performance was 38%. This was significantly above chance (16.66% given the 6-alternative forced-choice), $t(15) = 4.63$, $p<.001$. However, we saw no evidence of dependency; dependency in the data (D = 0.56) did not differ from the Independent model (D = 0.55), $t(15) = .31$, $p=.76$, and was significantly less than the Dependent model (D = 0.60), $t(15) = 3.74$, $p<.01$. Thus, although participants can sometimes deduce the correct answer for a non-encoded pair, presumably via the encoded within-event associations, their performance for non-encoded pairs was not dependent on performance for the encoded pairs. We speculate that the noisy and inaccurate process that allows performance to be above chance for the unseen association (e.g. B-C) does utilize the encoded associations (e.g. A-B, A-C), but does so via a process that itself introduces too much variation across events to be sensitive to “dependency” (i.e., co-variation with performance across events in the much better recall of A-B and A-C).

Further analyses

Yule's Q

To complement the modelling approach used in the main analyses, we further computed Yule's Q for the data for each contingency table and each individual participant. Yule's Q is a measure of correlation within a 2x2 contingency table, varying from +1 (complete dependence), through zero (independence), to -1 (negative dependence) (S2). For a 2x2 contingency table, where a = correct/correct, b = correct/incorrect, c = incorrect/correct and d = incorrect/incorrect, Yule's Q = [ad-bc]/[ad+bc]. It has previously been used to assess dependency between recognition and recall, A-B vs. A-C associations (see also experiment S2), and symmetric vs. asymmetric associations within an A-B pair (see S3–7).

Yule's Q is highly sensitive to the presence of a zero in the 2x2 contingency table (which forces Q to be 1, -1, or undefined). Given the high proportion of tables containing one of more zeros across all experiments (mean: 37%), the main analyses used a proportional measure (i.e., the proportion of counts on the leading diagonal of the contingency table, see Experimental Procedures), comparing proportional dependency between the data and the models. To get around this problem (but compromising the interpretation of the outcome), we added 0.5 to all cells for every contingency table prior to calculating Yule's Q for the data.

Experiment 1 revealed mean Yule's Q values of 0.46 (SD=0.30) (a relatively strong positive correlation) for the Simultaneous condition and 0.50 (SD=0.31) for the Separated condition (collapsed across Analysis-type and Item-type). In experiment 2, mean Yule's Q for the Separated Closed-loop condition was 0.34 (SD=0.26), however for the Separated Open-

loop condition it was 0.02 (SD=0.25). Thus, for the Simultaneous and Separated Closed-loop condition Yule's Q was relatively high, ranging between 0.35 and 0.50, whereas for the Open-loop condition it was close to zero. This same pattern was seen for experiment S1 (see Supplemental Experimental Procedures), with Yule's Q in the Separated Closed-loop condition 0.33 (SD=0.27) and Separated Open-loop condition -0.09 (SD=0.19) and in experiment S2, with Yule's Q in the Separated Closed-loop condition 0.41 (SD=0.32) and Separated Open-loop condition 0.06 (SD=0.38).

Inter-experimental analyses

One difference between the results of experiment S2 and experiments 1 and 2 and S1 is that we saw significantly less dependency in the data than the Dependent model in the Separated Closed-loop condition (though dependency was still greater than the Independent model). It is unclear why this may have occurred, although a similar result was also seen in Horner & Burgess (S1) where all events were presented simultaneously. As such, less dependency than the Dependent model is unlikely to be a function of the separated encoding conditions in experiment S2. To further investigate this possible difference we conducted a 2x4 (Data vs. Dependent model x Experiment) mixed ANOVA, focussing on the difference in dependency in the data and Dependent model across experiments 1,2, S1 and S2. This revealed a significant difference between the data and Dependent model, $F(1, 55) = 15.43$, $p<.001$, $\eta_P^2=.22$. No main effect of Experiment was seen, $F(3, 55) = 2.38$, $p=.08$, $\eta_P^2=.12$ (though we note a trend), nor was there an interaction between the two factors, $F(3, 55) = 0.77$, $p=.52$, $\eta_P^2=.04$.

Thus, the difference between the data and Dependent model did not vary systematically across experiments. When pooling data across experiments, we see less

dependency in the data than Dependent model. However, note that our main conclusions are based on significant differences in dependency between the Closed-loop and Open-loop conditions (relative to the baseline of the Independent model), and do not rest on the absence of difference between dependency in the data and Dependent model in the Closed-loop condition. Note also that the Dependent model acts as an upper bound for the amount of dependency present in the data. Any source of (independent) noise will decrease dependency in the data, relative to the Dependent model.

Supplemental Experimental Procedures

Experiment 1

Participants – Twenty participants (10 female) gave informed consent to participate. The mean age was 25.0 (SD = 4.4). Four were excluded due to poor performance across all conditions (<30% accuracy), leaving 16 participants (1 left handed).

Materials – Stimuli were 36 locations (e.g., a swimming pool), famous personalities (e.g., David Cameron) and common objects (e.g., a bicycle; see S1). Four randomised sets of events (i.e., location-person-object triads) were created and counterbalanced across participants.

Procedure – Experiments consisted of single study and test phases. At study, participants were serially presented with all 36 events. Half the events were seen in single encoding trials containing three elements (Simultaneous Closed-loop condition), whereas the other half were seen as three overlapping pairs of elements across three separate encoding trials (Separated Closed-loop condition). The order of pair presentation for each "event" in the Separated Closed-loop condition was randomised such that, across participants, all possible pair orders will have been seen in roughly equal proportions. This resulted in 72 encoding trials, 18 triads and 54 pairs, all of which were presented in interleaved fashion (Fig 1).

At test, participants were presented with an element and had to choose the associated element from six alternatives. All 36 events were tested with every cue-test pair (e.g., cue: location, test: object), resulting in 6 cued-recognition trials per event. One of the test items was the element associated with the cue. The other 5 elements were of the same category (e.g., objects) but were associated with other elements, randomly selected from

Simultaneous Closed-loop and Separated Closed-loop events. Participants were required to respond as accurately as possible within 6secs with a key-press, and following this rated their confidence on a scale of 1-5.

Assessing Dependency – We created 2x2 contingency tables of each participant's performance for specific pairs of associations (e.g., A-B and A-C) across events. We assessed dependency for retrieving two elements (e.g., person and object) when cued by the remaining within-event element (e.g., location; 'A_BA_C' analyses), as well as for retrieving one element (e.g., location) when cued by its associated elements (e.g., person and object; 'B_AC_A' analyses). This resulted in six 2x2 tables per participant per condition, one for each element (item-type) and analysis-type.

Each contingency table shows how performance retrieving one association from an event (e.g., retrieving B cued by A) depends on performance retrieving another element from that event (e.g., retrieving C cued by A). For any given participant, the mean proportion of correct retrievals of B (over *N* events) when cued by A is denoted by P_{AB}. We then created tables for an Independent model for each participant, where the probability of correctly or incorrectly retrieving two associations is simply the product of the mean probability for each association (i.e., the proportion of events for which both B and C were correctly retrieved when cued by A would be $P_{AB}P_{AC}$), see Table 2.

The Dependent model modifies the Independent model by weighting performance by an "episodic factor" (E^i) that varies across events. This factor captures the extent to which the probability of correctly retrieving the elements comprising a specific event differs from the average probability across all events. For example, when retrieving B cued by A for event *i*:

$$E^i_{AB} = (T^i_{BA}+T^i_{BC}+T^i_{CA}+T^i_{CB})/(P_{BA}+P_{BC}+P_{CA}+P_{CB}) \quad (1)$$

where, T^i_{BA}=1 if the participant correctly retrieves A when cued by B (otherwise, T^i_{BA}=0), and similarly for T^i_{BC} etc. Note that the episodic factor, for retrievals cued by A, is estimated from retrievals not cued by A (i.e., excluding T^i_{AB}, T^i_{AC}, P_{AB}, P_{AC}). Note also that we have previously shown the Dependent model is not significantly affected by whether E^i is calculated with or without retrieval trials relating to the same cue element (e.g., calculating E^i_{AB} only using T^i_{BC} and T^i_{CB} compared to only using T^i_{AB} and T^i_{BA} (see S1). The probability of correctly retrieving an association from event *i* is weighted by the episodic factor for that event, i.e., P_{AB} becomes $\acute{P}_{AB}=E^i_{AB}P_{AB}$. The Dependent model also takes into account the level of guessing, so that E^i weights the probability of deliberate correct retrieval but not the probability of guessing correctly (which should be independent of other responses). So the Dependent model follows the Independent model, with P^i_{AB} (and similarly P^i_{AC}) replaced by:

$$\acute{P}^i_{AB} = E^i_{AB}(P_{AB}- P_G/c)+ P_G/c \quad (2)$$

where P_G is the proportion of guesses, of which P_G/c will be correct in c-way forced choice cued-recognition. P_G is estimated as $c/(c$-1) times the proportion of errors. See Table 2 and (S1). The Independent model corresponds to setting E^i = 1 across all events.

The two models estimate the contingency tables corresponding to independent or dependent retrieval of elements within an event, controlling for overall accuracy and the level of guessing. To compare the data with the models we calculated a dependency measure based on the proportion of events where the retrieval of two associations is either both correct or both incorrect, where 1 = full dependency and 0.5 = full independence. Note that this dependency measure is modulated by accuracy. As such, only comparisons

between dependency in the data and the models (which explicitly control for level of accuracy) are meaningful. After analysing dependency across the A_BA_C and B_AC_A analyses and across Item-types (see Supplemental Results), we averaged across these factors for comparisons between the data and the models. We also calculated Yule's Q measure of dependency (see S2–4) for the data for all experiments (see Supplemental Results).

Experiment 2

Experiment 2 was identical to experiment 1 with the following exceptions.

Participants – Sixteen participants (10 female) gave informed consent to participate. Their mean age was 23.4 (SD = 3.6). One was excluded due to poor performance across all conditions (<30% accuracy), leaving 15 participants (1 left-handed).

Materials – A further 36 animals (e.g., a monkey) were included in the stimulus set to create 4-element open-loop events (Fig 1).

Procedure – All "events" were presented as three separate pairs across three separate encoding trials (as in experiment 1 Separated Closed-loop condition; Fig 1). For the Separated Closed-loop condition, half the events were location-person-object (L-P-O) triads and the other half location-person-animal (L-P-A) triads. For the L-P-O triads, the presentation order of the pairs was P-O, L-O, L-P. For the L-P-A triads the order was L-A, P-A, L-P. For the Separated Open-loop condition half the events were presented in the order P-A, L-O, L-P; and half in the order L-O, P-A, L-P. The final pair presented for both conditions was always the location-person association. Whereas in experiment 1 we analysed dependency for all combinations of pairs, in experiment 2 we only analysed dependency for the location and person elements (relative to the other associated elements), as this controls for item

familiarity (each person and location being presented twice in each condition, once in a person-location association and once in association with another type of element). The test phase was identical to Experiment 1, all pairs presented during the study phase were tested in both directions (e.g., retrieve person cued by location, as well as retrieve location cued by person).

Analyses – We report accuracy for all four elements across the Separated Closed-loop and Separated Open-loop conditions. Note, however, that event numbers vary for the animal and object elements in the Separated Closed-loop condition as half the events were L-P-O triads and half were L-P-A triads. Dependency was assessed only for associations including location and person. For the Closed-loop condition, we assessed dependency between retrieval of the object (or animal) and person when cued by the location, and for retrieval of the object (or animal) and location when cued by the person (A_BA_C analyses). We also assessed dependency between retrieval of the location cued by the object (or animal) and by the person, and retrieval of the person when cued by the object (or animal) and by the location (B_AC_A analyses).

For the Open-loop condition, we assessed dependency between retrieval of the object and person when cued by the location, and retrieval of the animal and location when cued by the person (A_BA_C analyses). We also assessed dependency between retrieval of the location when cued by the object and by the person, and of the person when cued by the animal and location (B_AC_A analyses). This resulted in 4 dependency measures per participant. Independent and Dependent models were constructed for each contingency table, as in experiment 1. We again averaged across these conditions for comparisons of dependency between the data and models (see Supplemental Results for analyses of dependency for the

data across these conditions).

Experiment S1

Experiment S1 was identical to experiment 2 with the following exceptions.

Participants – 15 participants (12 female) gave informed consent to participate. Participants had a mean age of 22.5 (SD = 4.3). One was excluded due to poor performance across all conditions (<30% accuracy), leaving 14 participants. By self-report, all participants were right-handed.

Procedure – Experiment S1 was designed to rule out possible order effects at encoding in experiment 2. Whereas in experiment 2 the 1st and 2nd encoded pairs for each Open-loop "event" were unrelated (e.g., location-object then person-animal), the 2nd pair in experiment S1 always overlapped with the first encoded pair in both the Closed- and Open-loop conditions. Four orders were used for both conditions. In the Closed-loop condition, pairs were seen in the orders: (1) P-O, L-P, L-O (2) L-A, L-P, P-A (3) L-P, L-O, P-O and (4) L-P, L-A, P-A. In the Open-loop condition, pairs were seen in the orders: (1) P-A, L-P, L-O (2) L-O, L-P, P-A (3) L-P, L-O, P-A and (4) L-P, P-A, L-O.

Experiment S2

Experiment S2 was identical to experiment 2 with the following exceptions.

Participants - 34 participants (19 female) gave informed consent to participate. 17 participants were assigned to the Separated Closed-loop and 17 participants to the Separated Open-loop condition. Participants had a mean age of 24.1 (SD = 4.1). Three were excluded from the Closed-loop group and one from the Open-loop group due to poor

performance across all conditions (<30% accuracy), leaving 14 participants in the Closed-loop group and 16 in the Open-loop group. By self-report, four participants were left-handed, the remainder right-handed.

Procedure - Half of the participants saw all three pairs of an event at encoding (the Closed-loop condition), resulting in 108 trials at Study. The other half saw only two out of the possible three pairs of an event at encoding (the Open-loop condition), resulting in 72 trials at Study. Of the 36 events in the Open-loop group, 12 events presented the location-person and location-object pairs, 12 the location-person and object-person pairs and 12 the location-object and object-person pairs. As in experiment 1, the presentation order of pairs for each "event" was randomised. At test, all pairs were tested (including the non-encoded pairs in the Open-loop group).

Analyses - The main analyses of the Open-loop events were restricted to those pairs that were encoded. Thus, we assessed accuracy and dependency only for pairs that the participant had actually seen. For the dependency analysis this meant both associations for a particular contingency table (e.g., the A-to-B vs. A-to-C associations) had to have been seen by the participants at encoding. Analysis of the Closed-loop events was identical to experiment 1.

Supplemental References

S1. Horner AJ, Burgess N (2013) The associative structure of memory for multi-element events. *J Exp Psychol Gen* 142:1370–1383.

S2. Bishop YM, Fienberg SE, Holland PW (1975) *Discrete multivariate analysis: theory and practice* (MIT Press, Cambridge, Mass.).

S3. Kahana MJ (2000) in *Oxford handbook of human memory*, eds Tulving E, Craik FIM (Oxford Press, England), pp 323–284.

S4. Kahana MJ, Rizzuto DS, Schneider AR (2005) Theoretical correlations and measured correlations: relating recognition and recall in four distributed memory models. *J Exp Psychol Learn Mem Cogn* 31:933–953.

S5. Kahana MJ (2002) Associative symmetry and memory theory. *Mem Cognit* 30:823–840.

S6. Caplan JB, Glaholt MG, McIntosh AR (2006) Linking associative and serial list memory: Pairs versus triples. *J Exp Psychol Learn Mem Cogn* 32:1244–1265.

S7. Caplan JB, Rehani M, Andrews JC Associations compete directly in memory. *Q J Exp Psychol*.
